# New Polyketides With Anti-Inflammatory Activity From the Fungus *Aspergillus rugulosa*

**DOI:** 10.3389/fphar.2021.700573

**Published:** 2021-06-21

**Authors:** Qianqian Xu, Yuben Qiao, Zijun Zhang, Yanfang Deng, Tianqi Chen, Li Tao, Qiaoxin Xu, Junjun Liu, Weiguang Sun, Ying Ye, Yuanyuan Lu, Changxing Qi, Yonghui Zhang

**Affiliations:** ^1^ Hubei Key Laboratory of Natural Medicinal Chemistry and Resource Evaluation, School of Pharmacy, Tongji Medical College, Huazhong University of Science and Technology, Wuhan, China; ^2^ Ezhou Central Hospital, Ezhou, China; ^3^ Maternal and Child Health Hospital of Hubei Province, Tongji Medical College, Huazhong University of Science and Technology, Wuhan, China

**Keywords:** *Aspergillus rugulosa*, polyketides, anti-inflammatory, molecular docking, immunofluorescence

## Abstract

Two new polyketide compounds, asperulosins A and B (**1**–**2**), and one new prenylated small molecule, asperulosin C (**3**), along with nine known compounds (**4**–**12**), were isolated and identified from a fungus *Aspergillus rugulosa*. Their structures were extensively elucidated *via* HRESIMS, 1D, and 2D NMR analysis. The absolute configurations of the new compounds were determined by the comparison of their electronic circular dichroism (ECD), calculated ECD spectra, and the detailed discussion with those in previous reports. Structurally, compounds **1** and **2** belonged to the polyketide family and were from different origins. Compound **2** was constructed by five continuous quaternary carbon atoms, which occur rarely in natural products. All of the isolates were evaluated for anti-inflammatory activity against the production of nitric oxide (NO) in lipopolysaccharide (LPS)-induced RAW264.7 cells. Among those, compounds **1** and **5** showed a significant inhibitory effect on NO production with IC_50_ values of 1.49 ± 0.31 and 3.41 ± 0.85 *μ*M, respectively. Additionally, compounds **1** and **5** markedly increased the secretion of anti-inflammatory cytokine IL10 while suppressing the secretion of pro-inflammatory cytokines IL6, TNF-α, IFN-γ, MCP-1, and IL12. Besides, **1** and **5** inhibited the transcription level of pro-inflammatory macrophage markers IL6, IL1β, and TNF-α while remarkably elevating the anti-inflammatory factor IL10 and M2 macrophage markers ARG1 and CD206. Moreover, **1** and **5** restrained the expression and nuclear translocation of NF-κB, as well as its downstream signaling proteins COX-2 and iNOS. All these results suggest that **1** and **5** have potential as anti-inflammatory agents, with better or comparable activities than those of the positive control, dexamethasone.

## Introduction

Small molecular polyketide compounds have been considered commercially valuable for their wide range of functions, structural diversity, and outstanding pharmaceutical activities (González-Medina et al., 2017; Newman and Cragg, 2020). For instance, lovastatin, daunomycin, and tetracycline are all small molecular polyketides (Herkommer et al., 2014; Van Wagoner et al., 2014). Natural polyketides are biosynthesized by consecutive decarboxylative condensations of short-chain acyl-CoAs by fungi, bacteria, and plants (Hertweck, 2009). Consequently, bioactive polyketides have roused extensive scientific interest in the fields of chemistry and pharmacology in recent years (Lacoske and Theodorakis, 2015).

Inflammation is a defensive response to fight infections such as microbial infection, chemical stimuli, and toxins (Medzhitov, 2010). However, recent studies have shown that uncontrolled chronic inflammation is associated with multiple diseases, including rheumatoid arthritis, metabolic syndrome, diabetes, and cancer (Okamoto et al., 2007). Overproduction of pro-inflammatory factors including nitric oxide (NO) and cyclooxygenase-2 (COX-2) and cytokines such as tumor necrosis factor-α (TNF-α) and interleukin-6 (IL-6) impulses the inflammatory response (Lee et al., 2016). Furthermore, the activation of the transcription factor nuclear factor-kappa B (NF-κB) accelerates the immune response *via* the transcriptional activation of the pro-inflammatory factors and cytokines mentioned above (Alvarez-Suarez et al., 2017).

**FIGURE 1:**
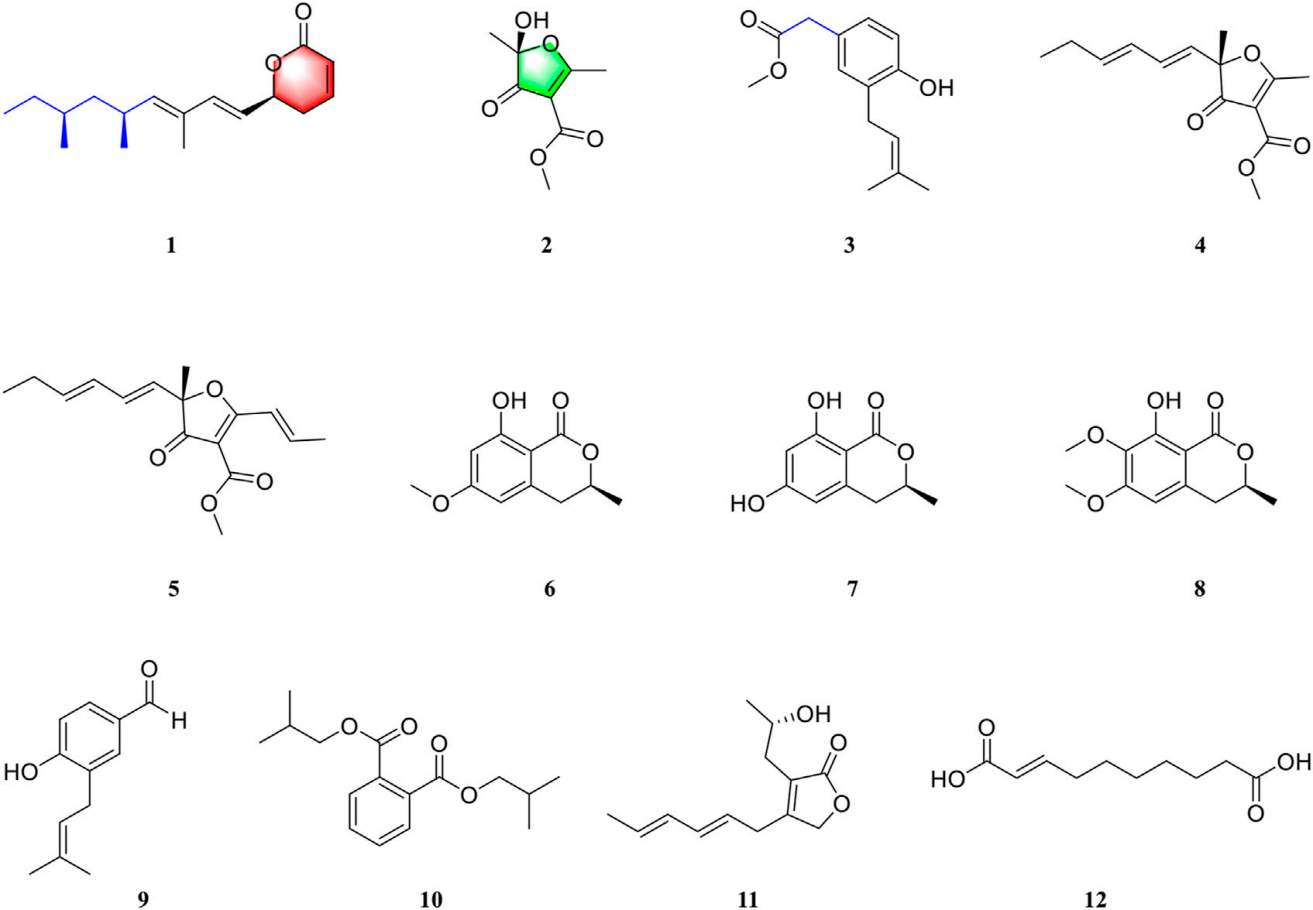
Structures of compounds **1**–**12**.

Our group focuses on the discovery and development of novel bioactive secondary metabolites from the *Aspergillus* species in recent years. Representative research studies include asperflavipine A (Zhu et al., 2017), epicochalasines A and B (Zhu et al., 2016), asperterpenes A and B (Qi et al., 2016), asperpyridone A (Qiao et al., 2019), and terreuspyridine (Li et al., 2020). Moreover, we recently found that large-scale culture can approach many more natural products with novel skeletons (Hu et al., 2021). As part of the program mentioned above, we performed a chemical investigation on a polyketide-producing fungus, *Aspergillus rugulosa* (Ballantine et al., 1969; Cacho et al., 2012). As a result, we isolated and identified two new polyketides, asperulosins A and B (**1**–**2**), and one new prenylated small molecule, asperulosin C (**3**), along with nine known compounds (**4**–**12**). These compounds were determined as (–)-gregatin B (**4**) (Burghart-Stoll and Brückner, 2012), aspertetronin A (**5**) (Burghart-Stoll and Brückner et al., 2012), angelicoin B (**6**) (Elsebai et al., 2018), (*R*)-6-hydroxymellein (**7**) (Islam et al., 2007), dimethoxymellein (**8**) (Choudhary et al., 2004), 4-hydroxy-3-(3-methylbut-2-enyl) benzaldehyde (**9**) (Vu et al., 2016), diisobutyl phthalate (**10**) (Zhang et al., 2003), 3-(2-hydroxypropyl)-4-(hexa-2*E*,4*E*-dien-6-yl) furan-2(5*H*)-one (**11**) (Almassi et al., 1991), and trans-2-decenedioic acid (**12**) (Kwok et al., 1992) by detailed comparison of their NMR data and specific rotations with those of previous literatures. Herein, the details of the isolation, structural elucidation, and biological evaluations of the isolates (Figure 1) are described.

## Materials and Methods

### General

Optical rotation, UV, and IR data were recorded on a PerkinElmer 341 instrument, a Varian Cary 50 instrument, and a Bruker Vertex 70 instrument with KBr pellets, respectively. ECD data were measured using a JASCO-810 CD spectrometer. The high-resolution electrospray ionization mass spectra (HRESIMS) were recorded by using the positive ion mode on a Thermo Fisher LC-LTQ-Orbitrap XL instrument. One- and two-dimensional NMR data were recorded on a Bruker AM-400 instrument, with the reference of ^1^H and ^13^C NMR chemical shifts of the solvent peaks for methanol-*d*_4_ (*δ*_H_ 3.31 and *δ*_C_ 49.0) and CHCl_3_-*d* (*δ*_H_ 7.26 and *δ*_C_ 77.0). Semi-preparative HPLC was conducted on a Dionex HPLC system equipped with an Ultimate 3,000 pump (Thermo Fisher Scientific, Germany), an Ultimate 3,000 autosampler injector, and an Ultimate 3,000 diode array detector (DAD) controlled by Chromeleon software (version 6.80), using a reverse-phased C18 column (5 *μ*m, 10 × 250 mm, Welch Ultimate XB-C18). Column chromatography (CC) was carried out by using silica gel (80–120, 100–200, 200–300 mesh, Qingdao Marine Chemical, Inc., Qingdao, People’s Republic of China), Lichroprep RP-C_18_ gel (40–63 *μ*m, Merck, Darmstadt, Germany), and Sephadex LH-20 (GE Healthcare Bio-Sciences AB, Sweden). Silica gel 60 F_254_ was used for the TLC (thin-layer chromatography) detection, and spots were visualized by spraying heated silica gel plates with 5% H_2_SO_4_ in EtOH.

### Fungal Material

The fungus *Aspergillus rugulosa* was purchased from the China General Microbiological Culture Collection Center (CGMCC, no. 3.6395). The fungal sample was deposited in the culture collection of Tongji Medical College, Huazhong University of Science and Technology.

### Fermentation, Extraction, and Purification

The experimental stain was incubated in potato dextrose agar (PDA) medium at 28 °C for 4 days to prepare the seed cultures, which was then transferred into 500-ml Erlenmeyer flasks, each containing 200 g rice (total 20 kg). After being cultivated for 21 days, the medium was extracted with 95% aqueous EtOH five times at room temperature. Afterward, the solvent was removed under reduced pressure to yield a total residue, which was then suspended in water and partitioned repeatedly with EtOAc. The EtOAc extract (130.4 g) was chromatographed by silica gel CC (80–120 mesh), using an increasing gradient of petroleum ether–ethyl acetate (100:0–0:100) to afford six fractions (A–F).

Fraction C (16.5 g) was fractioned by silica gel CC (200–300 mesh), using an increasing gradient of petroleum ether–ethyl acetate (20:1–10:1) to afford ten subfractions (C1–C10). Subfraction C4 (375 mg) was chromatographed on Sephadex LH-20 eluted with CH_2_Cl_2_–MeOH (1:1, v/v) to yield three fractions (C4.1–C4.3). Fraction C4.2 was purified by using semi-preparative HPLC eluted with MeCN–H_2_O (82:18, v/v, 2.0 ml/min) to afford compound **1** (*t*_R_ 15.2 min, 4.6 mg). Fraction C4.3 was purified by using semi-preparative HPLC (MeCN–H_2_O, 77:23, v/v, 2.0 ml/min) to yield compound **5** (*t*_R_ 15.8 min, 5.4 mg). Compound **9** (*t*_R_ 17.1 min, 3.0 mg) was purified by semi-preparative HPLC (MeCN–H_2_O, 52:48, v/v, 2.0 ml/min) from fraction C4.1. Subfraction C5 was chromatographed on Sephadex LH-20 eluted with CH_2_Cl_2_–MeOH (1:1, v/v) to yield five fractions (C5.1–C5.5). Fraction C5.2 was purified by using semi-preparative HPLC (MeOH–H_2_O, 80:20, v/v, 2.0 ml/min) to yield compound **10** (*t*_R_ 31.3 min, 18.2 mg). Fraction C5.3 was chromatographed using the RP-C_18_ column with MeOH–H_2_O (from 35:65 to 100:0, v/v) to afford ten subfractions (C5.3.1–C5.3.10). Compound **3** (*t*_R_ 25.1 min, 5.0 mg) was purified by semi-preparative HPLC (MeCN–H_2_O, 58:42, v/v, 2.0 ml/min) from fraction C5.3.2. Compound **2** (*t*_R_ 9.3 min, 4.9 mg) was purified by semi-preparative HPLC (MeCN–H_2_O, 30:70, v/v, 2.0 ml/min) from fraction C5.3.4. Compound **11** (*t*_R_ 41.3 min, 66.0 mg) was purified by semi-preparative HPLC (MeOH–H_2_O, 55:45, v/v, 2.0 ml/min) from fraction C5.3.5. Compound **4** (*t*_R_ 14.0 min, 55.6 mg) was purified by semi-preparative HPLC (MeOH–H_2_O, 79:21, v/v, 2.0 ml/min) from C5.3.6. Compound **12** (*t*_R_ 31.3 min, 3.5 mg) was purified by semi-preparative HPLC (MeOH–H_2_O, 75:45, v/v, 2.0 ml/min) from fraction C5.3.8.

Fraction E (1.4 g) was fractioned by silica gel CC (200–300 mesh), using an increasing gradient of petroleum ether–ethyl acetate (10:1–1:1) to afford five subfractions (E1–E5). Subfraction E4 (417 mg) was chromatographed using the RP-C_18_ column with MeOH–H_2_O (from 30:70 to 90:10, v/v) to afford eight subfractions (E4.1–E4.8). Compound **6** (*t*_R_ 19.2 min, 9.1 mg) was purified by semi-preparative HPLC (MeCN–H_2_O, 33:67, v/v, 2.0 ml/min) from fraction E4.3. Fraction E4.2 was purified by using semi-preparative HPLC (MeCN–H_2_O, 28:72, v/v, 2.0 ml/min) to afford compound **7** (*t*_R_ 16.3 min, 3.4 mg). Compound **8** (*t*_R_ 15.5 min, 16.4 mg) was purified by semi-preparative HPLC (MeCN–H_2_O, 47:53, v/v, 2.0 ml/min) from fraction E4.6.

### Spectroscopic Data

Compound **1**: colorless oil; (*α*)25 D: +2.0 (*c* 0.1, MeOH); UV (MeOH) *λ*_max_ (log *ε*) = 233 (3.34) nm; IR *ν*_max_ = 2,962, 1,725, 1,384, 1,345, 1,246, 1,028, 1,024, 818 cm^−1^; ECD (*c* 0.1, MeOH) Δ*ε*_202_—6.3, Δ*ε*_236_ + 0.20, Δ*ε*_258_—1.15; molecular formula C_17_H_26_O_2_; HRESIMS *m/z* 285.1837 (M + Na)^+^ (calcd for C_17_H_26_O_2_Na, 285.1830); for ^1^H and ^13^C NMR data, see Table 1.

**TABLE 1:** ^1^H (400 MHz) and ^13^C NMR (100 MHz) data for compounds **1**–**3** (*δ* in ppm, *J* in Hz).

No.	1 (in CDCl_3_)		No.	2 (in methanol-*d*_4_)		No.	3 (in CDCl_3_)	
	*δ*_H_ (*J* in Hz)	*δ*_C_, type		*δ*_H_ (*J* in Hz)	*δ*_C_, type		*δ*_H_ (*J* in Hz)	*δ*_C_, type
1	—	164.1 C	1	—	197.7 C	1	—	153.5 C
2	6.06 dt (9.8, 1.8)	121.6 CH	2	—	107.7 C	2	—	127.0 C
3	6.89 dt (9.8, 4.3)	144.7 CH	3	—	198.3 C	3	7.00 overlap	130.8 CH
4	2.47 m	30.1 CH_2_	4	—	164.4 C	4	—	126.0 C
5	4.97 ddd (15.0, 7.4, 0.8)	78.6 CH	5	—	106.4 C	5	6.74 dd (8.8, 7.9)	115.8 CH
6	5.61 dd (15.7, 6.8)	122.2 CH	6	1.49 s	21.8 CH_3_	6	7.02 overlap	128.2 CH
7	6.34 d (15.7)	138.9 CH	7	2.60 s	18.3 CH_3_	7	3.53 s	40.4 CH_2_
8	—	130.7 C	8	3.78 s	51.6 CH_3_	8	—	172.5 C
9	5.29 d (9.8)	142.4 CH	—	—	—	9	3.33 br d (7.4)	29.8 CH_2_
10	2.59 m	30.3 CH	—	—	—	10	5.30 tq (7.3, 2.8)	121.6 CH
11*α*	1.27 overlap	44.7 CH_2_	—	—	—	11	—	134.8 CH
11*β*	1.09 overlap		—	—	—	12	1.77 s	17.9 CH_3_
12	1.25 overlap	32.2 CH	—	—	—	13	1.77 s	25.8 CH_3_
13*α*	1.27 overlap	30.0 CH_2_	—	—	—	14	3.69 s	52.0 CH_3_
13*β*	1.13 overlap		—	—	—	—	—	—
14	0.85 d (7.1)	11.3 CH_3_	—	—	—	—	—	—
15	1.75 d (0.8)	12.5 CH_3_	—	—	—	—	—	—
16	0.94 d (6.7)	21.4 CH_3_	—	—	—	—	—	—
17	0.81 d (6.5)	19.1 CH_3_	—	—	—	—	—	—

Compound **2**: white powders; (*α*)25 D: 52.2 (*c* 0.1, MeOH); IR *ν*_max_ = 3,425, 1,732, 1,649, 1,579, 1,447, 1,205, 1,009 cm^−1^; ECD (*c* 0.1, MeOH) Δ*ε*_214_ + 8.37, Δ*ε*_241_ − 2.62, Δ*ε*_268_ + 3.78; HRESIMS *m/z* 209.0404 (M + Na)^+^ (calcd for C_8_H_10_O_5_Na, 209.0426); for ^1^H and ^13^C NMR data, see Table 1.

Compound **3**: white powders; UV (MeOH) *λ*_max_ (log *ε*) = 202 (4.16), 279 (4.01) nm; IR *ν*_max_ = 3,429, 1,720, 1,613, 1,588, 1,438, 1,265, 1,018 cm^−1^; molecular formula C_14_H_18_O_3_, HRESIMS *m/z* 257.1155 (M + Na)^+^ (calcd for C_14_H_18_O_3_Na, 257.1154); for ^1^H and ^13^C NMR data, see Table 1.

Compound **4** [(–) gregatin B]: Colorless oil; (*α*)25 D = −178.0 (MeOH); ^1^H-NMR (400 MHz, CDCl_3_) *δ*_H_: 0.98 (3H, t, *J* = 7.5 Hz, H_3_-6′′), 1.52 (3H, s, H_3_-2), 2.09 (2H, q, *J* = 7.5 Hz, H_2_-5′′), 2.64 (3H, s, H_3_-6), 3.82 (3H, s, OCH_3_-1′), 5.53 (1H, d, *J* = 15.5 Hz, H-1′′), 5.81 (1H, dt, *J* = 15.5, 6.5 Hz, H-4′′), 5.96 (1H, m, H-3′′), and 6.27 (1H, dd, *J* = 15.5, 10.3 Hz, H-2′′).

Compound **5** (aspertetronin A): Colorless oil; ^1^H-NMR (400 MHz, CDCl_3_) *δ*_H_: 0.98 (3H, t, *J* = 7.5 Hz, H_3_-6′), 1.54 (3H, s, H_3_-7′), 2.07 (2H, m, H_2_-5′), 3.83 (3H, s, OCH_3_-1‴), 5.56 (1H, d, *J* = 15.5 Hz, H-1′), 5.80 (1H, dt, *J* = 15.5, 6.5 Hz, H-4′), 5.96 (1H, m, H-3′), 6.26 (1H, dd, *J* = 15.5, 10.3 Hz, H-2′), 7.21 (1H, m, H-2′′), and 7.33 (1H, m, H-1′′). ^13^C-NMR (100 MHz, CDCl_3_) *δ*_C_: 13.3 (C-6′), 19.4 (C-3′′), 22.5 (C-7′), 26.7 (C-5′), 51.6 (OCH_3_-1‴), 90.4 (C-5), 103.7 (C-3), 120.8 (C-1′′), 126.1 (C-1′), 127.7 (C-3′), 131.5 (C-2′), 139.3 (C-4′), 144.8 (C-2′′), 163.9 (C-1‴), 185.2 (C-2), and 197.0 (C-4).

Compound **6** (angelicoin B): White amorphous powder; ^1^H-NMR (400 MHz, CDCl_3_) *δ*_H_: 1.47 (3H, d, *J* = 6.3 Hz, CH_3_-3), 2.82 (2H, br d, *J* = 7.3 Hz, H_2_-4), 3.78 (3H, s, OMe-6), 3.90 (3H, s, OMe-7), 4.63 (1H, m, H-3), 6.21 (1H, d, *J* = 2.2 Hz, H-5), 6.31 (1H, d, *J* = 2.3 Hz, H-7), and 11.2 (1H, s, OH-8). ^13^C-NMR (100 MHz, CDCl_3_) *δ*_C_: 20.6 (C-9), 34.7 (C-4), 55.4 (OMe-6), 75.4 (C-3), 99.3 (C-7), 101.4 (C-8a), 106.0 (C-5), 140.9 (C-4a), 164.3 (C-8), 165.6 (C-6), and 169.8 (C-1).

Compound **7** [(*R*)-6-hydroxymellein]: White amorphous powder; ^1^H-NMR (400 MHz, MeOH-*d*_4_) *δ*_H_: 1.46 (3H, d, *J* = 6.3 Hz, CH_3_-3), 2.82 (1H, dd, *J* = 16.4, 11.0 Hz, H-4a), 2.91 (1H, dd, *J* = 16.4, 3.7 Hz, H-4b), 4.66 (1H, m, H-3), 6.19 (1H, d, *J* = 2.3 Hz, H-5), and 6.21 (1H, d, *J* = 2.3 Hz, H-7). ^13^C-NMR (100 MHz, MeOH-*d*_4_) *δ*_C_: 20.9 (C-9), 35.5 (C-4), 71.2 (C-3), 101.3 (C-7), 102.2 (C-8a), 107.9 (C-5), 143.5 (C-4a), 165.7 (C-8), 166.5 (C-6), and 171.7 (C-1).

Compound **8** [(–)-(3*R*)-6,7-Dimethoxymellein]: Colorless crystalline solid; ^1^H-NMR (400 MHz, CDCl_3_) *δ*_H_: 1.51 (3H, d, *J* = 6.3 Hz, H_3_-9), 2.86 (2H, m, H_2_-4), 3.87 (3H, s, OMe-6), 3.90 (3H, s, OMe-7), 4.66 (1H, m, H-3), 6.28 (1H, s, H-5), and 11.1 (1H, s, OH-8). ^13^C-NMR (100 MHz, CDCl_3_) *δ*_C_: 20.6 (C-9), 34.6 (C-4), 56.1 (OMe-6), 60.7 (OMe-7), 75.8 (C-3), 102.0 (C-5), 102.8 (C-8a), 135.4 (C-7), 156.1 (C-8), 158.4 (C-6), and 169.8 (C-1).

Compound **9** [4-hydroxy-3-(3-methylbut-2-enyl) benzaldehyde]: Colorless oil; ^1^H-NMR (400 MHz, MeOH-*d*_4_) *δ*_H_: 1.72 (3H, s, H_3_-4′), 1.76 (3H, s, H_3_-5′), 3.33 (2H, overlap, H_2_-1′), 5.34 (1H, ddq, *J* = 8.9, 5.9, 1.5 Hz, H-2′), 6.89 (1H, d, *J* = 8.2 Hz, H-5), 7.60 (1H, dd, *J* = 8.2, 2.3 Hz, H-6), 7.62 (1H, d, *J* = 2.3 Hz, H-2), and 9.72 (1H, s, CHO). ^13^C-NMR (100 MHz, MeOH-*d*_4_) *δ*_C_: 17.8 (C-4′), 25.9 (C-5′), 28.9 (C-1′), 116.0 (C-2, C-5), 122.9 (C-2′), 130.2 (C-3), 130.4 (C-1), 131.4 (C-5), 132.3 (C-6), 134.1 (C-3′), 163.0 (C-4), and 193.2 (C-7).

Compound **10** (diisobutyl phthalate): Colorless viscous liquid; ^1^H-NMR (400 MHz, MeOH-*d*_4_) *δ*_H_: 0.99 (12H, d, *J* = 6.7 Hz, 4 × CH_3_), 2.03 (2H, dp, *J* = 13.4, 6.7 Hz), 4.07 (4H, d, *J* = 6.5 Hz), 7.62 (2H, dd, *J* = 5.7, 3.3 Hz), and 7.73 (2H, dd, *J* = 5.7, 3.3 Hz). ^13^C-NMR (100 MHz, MeOH-*d*_4_) *δ*_C_: 19.5, 29.0, 72.9, 129.9, 132.3, 133.6, and 169.2.

Compound **11** [3-(2-hydroxypropyl)-4-(hexa-2*E*,4*E*-dien-6-yl) furan-2(5*H*)-one]: Colorless oil; ^1^H-NMR (400 MHz, MeOH-*d*_4_) *δ*_H_: 1.18 (3H, d, *J* = 6.2 Hz, H_3_-3′′), 1.73 (3H, br d, *J* = 6.8 Hz, H_3_-1′), 2.40 (2H, m, H_2_-1′′), 3.28 (2H, d, *J* = 7.1 Hz, H_2_-6′), 3.96 (1H, m, H-2′′), 4.75 (2H, s, H_2_-5), 5.55 (1H, dt, *J* = 14.0, 7.1 Hz, H-5′), 5.68 (1H, dq, *J* = 13.7, 6.8 Hz, H-2′), 6.05 (1H, m, H-3′), and 6.15 (1H, m, H-4′). ^13^C-NMR (100 MHz, MeOH-*d*_4_) *δ*_C_: 18.1 (C-1′), 23.3 (C-3′′), 31.2 (C-6′), 34.1 (C-1′′), 67.0 (C-2′′), 73.2 (C-5), 124.8 (C-5′), 125.6 (C-4), 130.1 (C-2′), 132.1 (C-3′), 135.1 (C-4′), 164.3 (C-3), and 177.7 (C-2).

Compound **12** (*trans*-2-decenedioic acid): Colorless oil; ^1^H-NMR (400 MHz, MeOH-*d*_4_) *δ*_H_: 1.36 (2H, m, H_2_-5), 1.36 (2H, m, H_2_-6), 1.48 (2H, m, H_2_-7), 1.60 (2H, m, H_2_-8), 2.22 (2H, m, H_2_-4), 2.28 (2H, m, H_2_-9), 5.80 (1H, d, *J* = 15.6 Hz, H-2), and 6.94 (1H, dt, *J* = 15.5, 7.0 Hz, H-3). ^13^C-NMR (100 MHz, MeOH-*d*_4_) *δ*_C_: 26.0 (C-8′), 29.1 (C-7), 29.9 (C-5), 30.0 (C-6), 33.0 (C-4), 35.0 (C-9), 122.7 (C-2), 150.9 (C-3), 170.3 (C-1), and 177.7 (C-10).

### Cell Culture and Administration

Mouse macrophage-like cell line RAW 264.7 was cultured at 37 °C in a 5% CO_2_ environment in DMEM (Hyclone, United States) supplemented with 10% fetal calf serum (Gibico, United States), the antibiotics of penicillin/streptomycin (100 units/ml) (Invitrogen, United States), and 1.5% horse serum. LPS (1 μg/ml) was used to activate RAW264.7 cells. Dexamethasone (DEX) was used as the positive control.

### Measurement of Nitric Oxide (NO) and Cytotoxic Assay

RAW 264.7 cells (5 × 10^4^ cells/ml) were seeded in 96-well plates in 100 μl culture. After preincubation for 24 h, the seeded cells were treated with tested compounds ranging from 40 to 5 *µ*M for 1 h, followed by stimulation with LPS (1 μg/ml) for another 24 h. The production of NO was determined with Griess reagent. Cell culture supernatants (50 μl) were mixed with 100 μl Griess reagent (1% sulfanilamide/0.1% naphthylethylene diamine dihydrochloride/2% phosphoric acid) in a 96-well plate for 10 min at room temperature. Then, the optical density was measured at 510 nm using a microplate reader (Thermo Fisher Scientific, United States). After 24 h of treatment with tested compounds, 10 μl of CCK-8 solution was added to each well, and the cells were incubated for a further 2 h, followed by the detection of absorbance at 450 nm.

### Cytokine Production Bioassay

Cells were treated as described above. After 24 h of treatment, the secretion level of cytokines IL6, IL10, MCP-1, IFN-γ, TNF-α, and IL12 in the culture supernatant was detected using an Ms Inflammation Cytometric Bead Array (CBA) kit (BD Pharmingen, United States) according to the manufacturer’s protocol.

### Quantitative Real Time Polymerase Chain Reaction Tests

RNA was isolated from RAW264.7 cells treated with the tested compounds (Invitrogen, Thermo Fisher Scientific, United States) according to the manufacturer’s recommendations. Total RNA was reverse-transcribed into cDNA using a transcription kit (ABP, United States). Quantitative RT-PCR (qRT-PCR) was performed using SYBR Green qPCR Mix (ABP, United States) with 0.2-μM forward and reverse primers in a final volume of 10 μl, and detection was performed using ABI QuantStudio 5 (Thermo Fisher Scientific, United States). The resulting cDNA was amplified by incubating at 95 °C for 5 min, 40 cycles of denaturation at 95 °C for 10 s, annealing at 55–60 °C for 20 s, and extension at 72 °C for 30 s. Values were exhibited relative to β-actin. The corresponding primer sequences are listed in Supplementary Table S1.

### Western Blot Analysis

Total proteins from the RAW264.7 cells were lysed in radioimmunoprecipitation assay (RIPA, Beyotime, China) buffer, and 30 μg total proteins were used for each blot. The samples were separated by SDS-PAGE and transferred onto a nitrocellulose filter (NC, Millipore, United States) membrane by electro-blotting. The membranes were blocked for 1 h and then incubated overnight with 1:1,000 dilutions of anti-TLR4, anti-iNOS, anti-COX2, and anti–NF-κB p65 (Cell Signaling Technology, United States). After incubation with the secondary antibody anti-mouse IgG (H + L) (DyLight™ 800, Cell Signaling Technology, United States) at 1:15,000 dilutions, the membranes were imaged using a LiCor Odyssey scanner (LI-COR, United States). Protein expressions were normalized using β-actin as the reference (Cell Signaling Technology, United States) in the same sample.

### Immunofluorescence

The cell-seeded glass cover slips were fixed with 4% cold paraformaldehyde for 15 min and permeabilized with 0.1% Triton X-100 for 30 min. Then, the cover slips were blocked with 5% BSA for 1 h and incubated with a primary antibody specific to the NF-κB p65 subunit (Cell Signaling Technology, United States) overnight at 4°C, followed by a secondary antibody labeled with Alexa Fluor-594 (1:5,000) for 1 h at room temperature, protected from light. After being stained with DAPI (5 μg/ml in PBS) for 30 min at 37 °C, the cover slips were washed and sealed. Images were obtained using an OLYMPUS IX73 fluorescence microscope (Olympus, Tokyo, Japan) with excitation/emission wavelengths of 590 nm/617 nm for Alexa Fluor-594 and 360 nm/450 nm for DAPI, respectively.

### Molecular Docking

The virtual docking was carried out in the Surflex-Dock module of FlexX/Sybyl software, which belongs to a fast docking method that allows sufficient flexibility of ligands and keeps the target protein rigid. Molecules were built using Chemdraw software and further optimized at the molecular, mechanical, and semiempirical level using Open Babel GUI. The crystallographic ligands were extracted from the active site, and the designed ligands were modeled. All the hydrogen atoms were added to define the correct ionization and tautomeric states, and the carboxylate, phosphonate, and sulfonate groups were considered in their charged form. In the docking calculation, the default FlexX scoring function was applied for exhaustive searching, solid body optimizing, and interaction scoring. Finally, the ligands with the lowest energy and the most optimum orientation were chosen.

### Statistical Analysis

All experiments were conducted in biological triplicate. All data are displayed as the mean ± standard error of the mean (SEM). Differences were evaluated by the Student’s *t*-test using GraphPad Prism software (GraphPad Prism version 5.01 for Windows, San Diego, CA). Differences with *p and ^#^ *p* < 0.05 were considered statistically significant.

## Results

### Structure Elucidation

**FIGURE 2:**
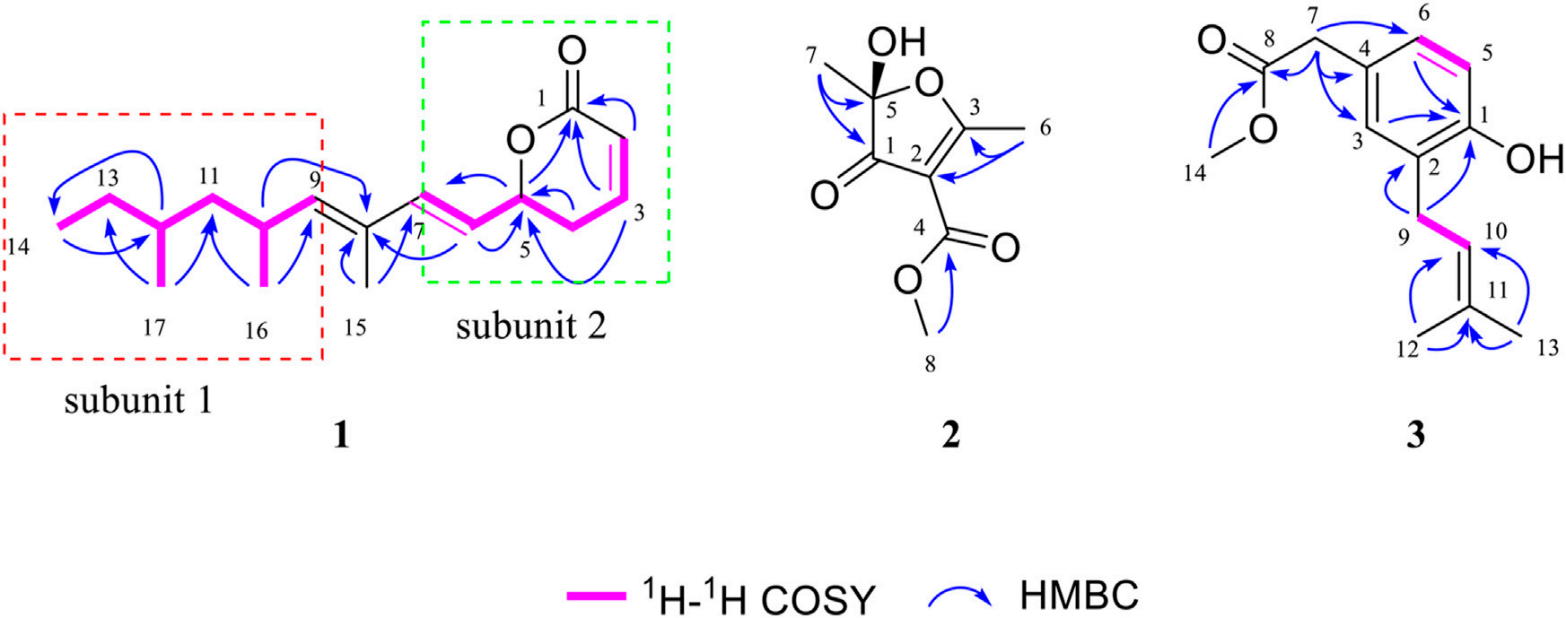
Selected ^1^H–^1^H COSY and HMBC correlations of **1**–**3**.

Compound **1** was isolated as colorless oil. The molecular formula C_17_H_26_O_2_ was deduced from the HRESIMS data (Supplementary Figure S7) at *m/z* 285.1837 (M + Na)^+^ (calcd for C_17_H_26_O_2_Na, 285.1830), referring to five degrees of unsaturation. The ^13^C NMR and DEPT spectroscopic data (Table 1; Supplementary Figure S2) of **1** revealed 17 carbon resonances that were attributed to four methyls at *δ*_C_ 11.3 (C-14), 12.5 (C-15), 21.4 (C-16), and 19.1 (C-17), three sp^3^ methylenes at *δ*_C_ 30.1 (C-4), 44.7 (C-11), and 30.0 (C-13), five olefinic methines at *δ*_C_ 121.6 (C-2), 144.7 (C-3), 122.2 (C-6), 138.7 (C-7), and 142.4 (C-9), three sp^3^ methines at *δ*_C_ 78.6 (C-5), 30.3 (C-10), and 32.2 (C-12), one olefinic quaternary carbon at *δ*_C_ 130.7 (C-8), and one ester carbonyl at *δ*_C_ 164.1 (C-1). The ^1^H NMR spectrum (Table 1; Supplementary Figure S1) of **1** showed diagnostic signals for four methyls, three sp^3^ methylenes, five olefinic methines, and three sp^3^ methines, which were attributed to the corresponding carbon atoms with the help of the HSQC spectrum. One carbonyl group and six olefinic carbon atoms, accounting for 4 out of 5 degrees of unsaturation, indicated that **1** possessed a monocyclic ring system. The key ^1^H–^1^H COSY spin–spin coupling system (Figure 2; Supplementary Figure S5) of H-9/H-10/H_2_-11/H-12/H_2_-13/H_3_-14, H_3_-16/H-10, and H_3_-17/H-12 in **1** indicated the direct carbon–carbon connectivity of C-9/C-10/C-11/C-12/C-13/C-14, C-16/C-10, and C-17/C-12. Subunit 1 was deduced and confirmed by the key HMBC correlations (Figure 2; Supplementary Figure S4) from H_3_-16 to C-9 and C-11, from H_3_-17 to C-11 and C-13, from H_3_-14 to C-12, and from H-12 to C-14. In the same manner, the direct carbon–carbon connectivity of C-2/C-3/C-4/C-5/C-6/C-7 was elucidated. The obvious HMBC correlations from H-2 (*δ*_H_ 6.06 dt *J* = 9.8, 1.8 Hz), H-3 (*δ*_H_ 6.89 dt *J* = 9.8, 4.3 Hz), and H-5 (4.97 ddd *J* = 15.0, 7.4, 0.8 Hz) to C-1 (*δ*_C_ 164.1) and from H-3 and H_2_-4 (*δ*_H_ 2.47 m) to C-5 (*δ*_C_ 78.6) showed the presence of lactone groups in subunit 2. The construction of subunits 1 and 2 was connected by an olefinic quaternary carbon atom (*δ*_C_ 130.7, C-8), which was deduced from the key HMBC cross peaks (Figure 2) of H-6/C-8, H-10/C-8, H_3_-15/C-8, and H_3_-15/C-7. Thus, the planar structure of **1** was determined (Figure 2).

**FIGURE 3:**
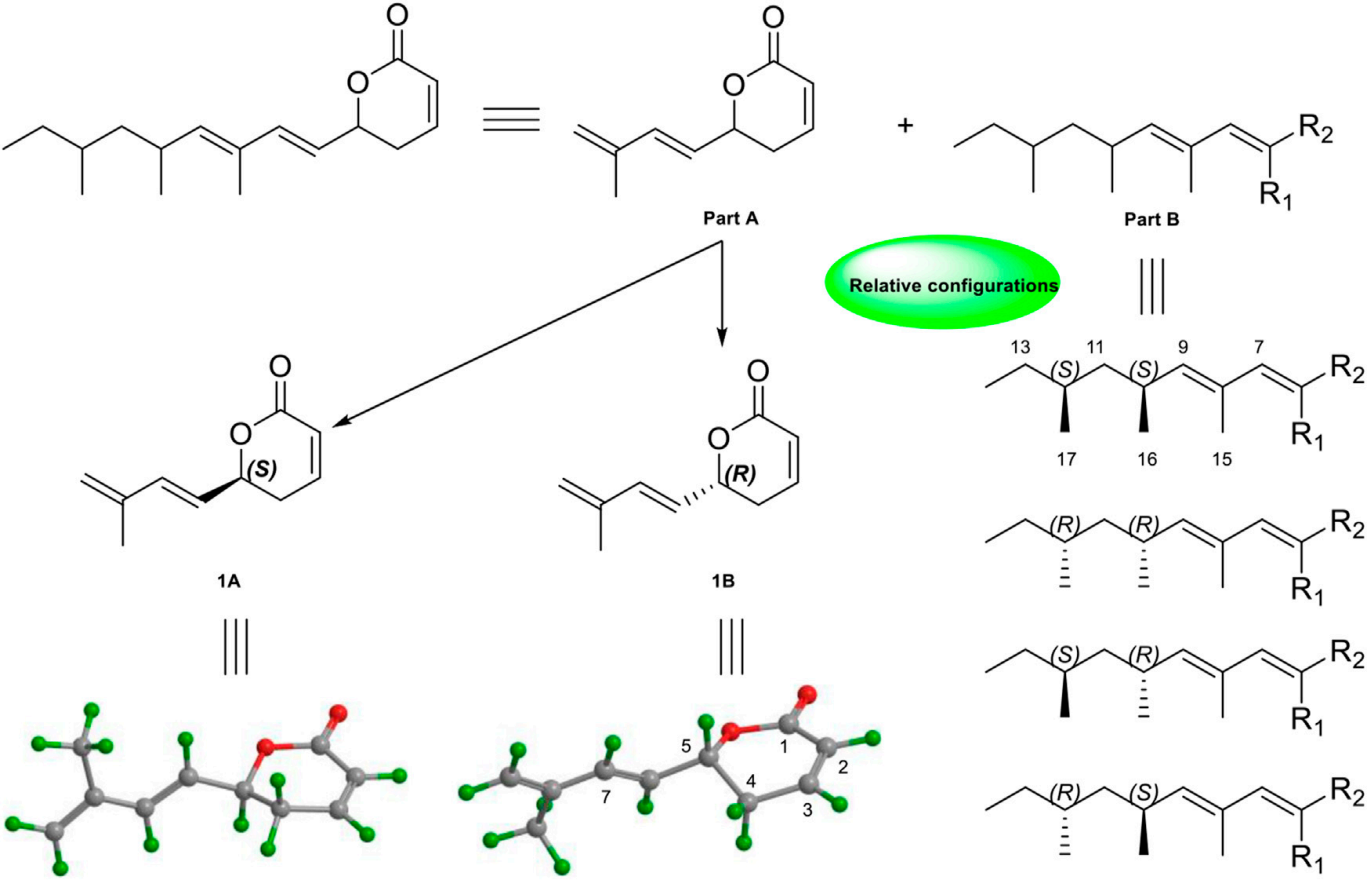
Key NOESY correlations and relative configurations of compound **1.**

**FIGURE 4:**
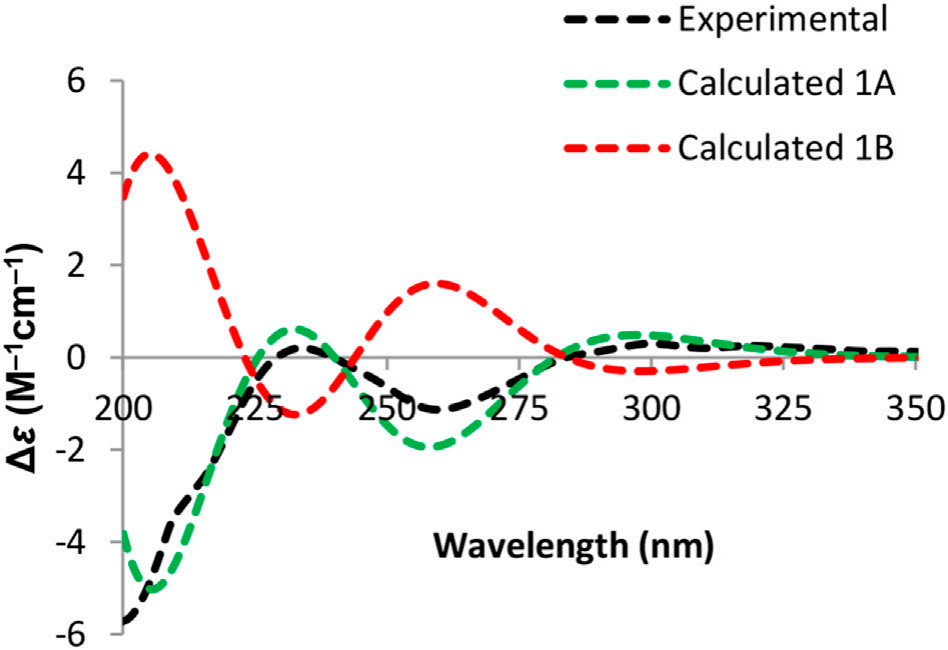
Experimental and calculated ECD spectra of **part A**.

Due to the presence of side chains in **1**, its relative configuration was determined with difficulty. In order to search for evidence for the configurational elucidation, we disassembled the structure of **1** into parts A and B (Figure 3). The configurations of **1A** (5*S*) and **1B** (5*R*) were calculated *via* the time-dependent density functional theory (TDDFT) method at the B3LYP/6-311++G** level with PCM in MeOH (Qiao et al., 2016; Qiao et al., 2019). In this case, the experimental curve displayed a good accordance behavior with the calculated ECD of **1A** and showed an absolutely reverse curve to that of enantiomer **1B** (Figure 4). Thus, the absolute configuration of part A was elucidated as 5*S*. Raffaele Riccio and co-workers (Bifulco et al., 2007) reported a useful method for the determination of relative configuration in organic compounds. In the rule, 16*β* is determined as the chemical shift of C-15 at less or more than 12.0 ppm (Table 2). Accordingly, the 16*β* of **1** was established because of the chemical shift of C-15 at *δ*_C_ 12.5 ppm. Moreover, the chemical shifts of C-13 (*δ*_C_ 30.0), C-16 (*δ*_C_ 21.4), and C-17 (*δ*_C_ 19.1), located in the intervals of 30.0–30.5, 21.0–21.6, and 18.9–19.2 ppm, respectively, showed the agreement with the configurations of 16*β* and 17*β* (Table 2). The discussions mentioned above enable us to elucidate the absolute configuration of **1** as 5*S*, 16*S*, and 17*S*. Using the search network SciFinder (https://scifinder.cas.org), compound **1** was determined as a unique linear chain compound with an *α, β*-unsaturated monocyclic lactone structure originating from the polyketide family.

**TABLE 2:** Previously reported rule for the identification of relative configurations in side chain compounds (Bifulco et al., 2007).

Position	16*α*, 17*α*	16*α*, 17*β*	16*β*,17*α*	16*β*,17*β*
Chemical shift (ppm)	C13, 30.0–31.0	C13, 29.0–29.3	C13, 28.9–29.5	C13, 30.0–30.5
	C15 < 12.0	C15 < 12.0	C15 > 12.0	C15 > 12.0
	C16, 21.0–21.6	C16, 20.2–20.5	C16, 20.5–20.8	C16, 21.0–21.6
	C17 < 18.5	C17, 19.0–19.6	C17, 19.0–19.6	C17, 18.9–19.2

Compound **2** was obtained as a white, amorphous powder and showed a molecular formula of C_8_H_10_O_5_, on the basis of its HRESIMS analysis (Supplementary Figure S14) at *m/z* 209.0404 (M + Na)^+^ (calcd for C_8_H_10_O_5_Na, 209.0426), requiring four degrees of unsaturation. The IR spectrum (Supplementary Figure S15) of **2** exhibited characterized absorption bands for hydroxy groups (3,425 cm^−1^) and ester/lactone carbonyl groups (1732 cm^−1^). The ^1^H NMR spectrum (Table 1; Supplementary Figure S10) of **2** showed two sp^3^ methyls [(*δ*_H_ 1.49, s, CH_3_-6) and (*δ*_H_ 2.60, s, CH_3_-7)] and one methoxy group at *δ*_H_ 3.78 (s, CH_3_-8) signals. The ^13^C NMR data (Table 1; Supplementary Figure S11) of **2** were attributed to one carbonyl group (*δ*_C_ 197.7, C-1), one lactone carbonyl group (*δ*_C_ 164.4, C-4), two sp^2^ quaternary carbons (*δ*_C_ 107.7, C-2 and *δ*_C_ 198.3, C-3), and one hemiacetal carbon atom at *δ*_C_ 106.4 (C-5). The key HMBC correlations (Supplementary Figure S13) from H_3_-6 to C-2 and C-3, from H_3_-7 to C-1 and C-5, and from the methoxy proton (*δ*_H_ 3.78) to C-4, coupled with the molecular degrees of unsaturation, deduced the planar structure of **2**, as shown in Figure 2.

**FIGURE 5:**
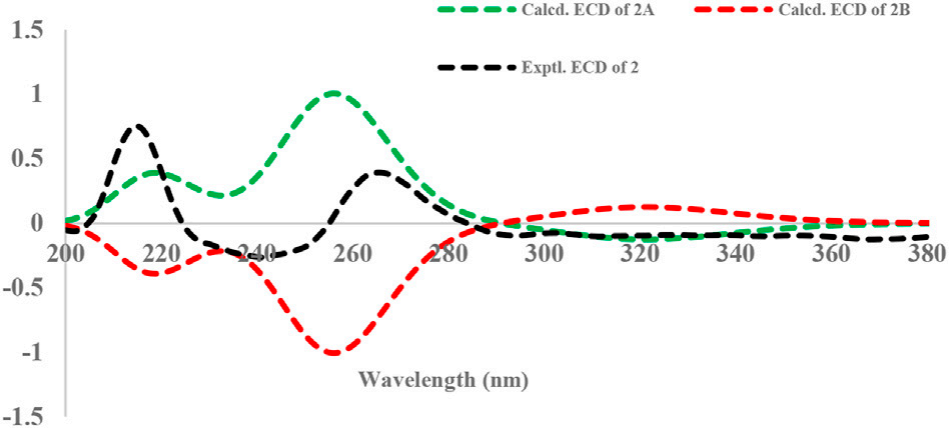
Experimental and calculated ECD spectra of **2.**

To determine the absolute configuration of C-5, the time-dependent density functional theory (TDDFT) method at the B3LYP/6-311++G** level with PCM in MeOH was performed for **2A** (5*S*) and **2B** (5*R*). The calculated ECD spectrum of **2A** matched well with that of the experimental ECD (Figure 5), indicating that the absolute configuration of **2** was elucidated as 5*S*.

Compound **3** was obtained as a white, amorphous powder, and the molecular formula C_14_H_18_O_3_ was established according to HRESIMS (Supplementary Figure S21) *m/z* 257.1155 (M + Na)^+^ (calcd for C_14_H_18_O_3_Na, 257.1154), requiring six degrees of unsaturation. The ^1^H NMR and ^13^C NMR spectra (Table 1; Supplementary Figures S16, S17) exhibited signals indicating the presence of a 1,2,4-trisubsituted phenyl group with the NMR data C-1 (*δ*_C_ 153.5), C-2 (*δ*_C_ 127.0), CH-3 (*δ*_H_ 7.00 overlap, *δ*_C_ 130.8), C-4 (*δ*_C_ 126.0), CH-5 (*δ*_H_ 6.74 dd, *J* = 8.8, 7.9 Hz, *δ*_C_ 115.8), and CH-6 (*δ*_H_ 7.02 overlap, *δ*_C_ 128.2). The key ^1^H–^1^H COSY spin–spin coupling system (Figure 2; Supplementary Figure S20) of H_2_-9/H-10 and the HMBC (Supplementary Figure S19) correlations from H_3_-12 and H_3_-13 to C-10 and C-11 (Figure 2) indicated the presence of a classical isopentenyl group. Additionally, the key HMBC correlations from H_2_-9 to C-1 and C-2 and from H-3 and H-6 to C-1 indicated the isopentenyl group locating at C-2. In turn, the HMBC correlations from H_3_-14 and H_2_-7 to C-8 and from H_2_-7 to C-3, C-4, and C-6 constructed a methyl acetate group connected to the phenyl group locating at C-4. Moreover, the chemical shift of C-1 (*δ*_C_ 153.5), coupled with HRESIMS, indicated a hydroxyl group locating at C-1. The chemical structure was thus elucidated as shown in Figure 1.

### One and Five Suppressed LPS-Induced Nitric Oxide Production

**FIGURE 6:**
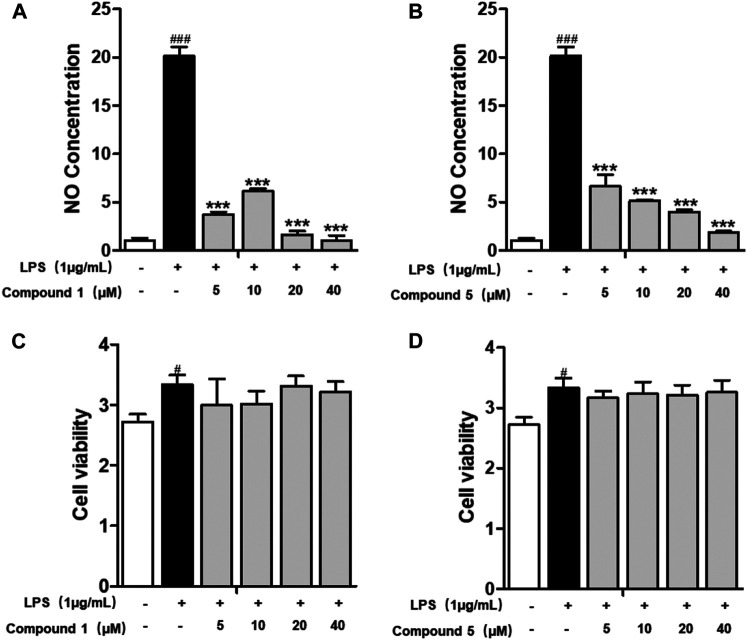
Compounds **1** and **5** exert significant anti-inflammatory effect on RAW264.7 macrophage cells. Effects of **1** and **5** on the NO production **(A, B)** and cell viability **(C, D)** of LPS-induced RAW264.7. Cells were treated with various concentrations of **1** and **5** ranging from 5, 10, 20, and 40 *μ*M. Data were presented as the mean ± SEM (*n* = 4). #*p* < 0.05, ###*p* < 0.001 vs. control, ***p < 0.001 vs. model.

All of the isolates were evaluated for anti-inflammatory activity against the production of NO in RAW264.7 stimulated by LPS. Among the isolates evaluated, compounds **1** and **5** showed a significant inhibitory effect on NO production, with IC_50_ values of 1.49 ± 0.31 and 3.41 ± 0.85 *μ*M, respectively, while the others exhibited no obviously inhibitory activities, with IC_50_ values up to over 40 *μ*M (Figures 6A,B; Table 3). Besides, the cytotoxicity assay exhibited that no apparent toxicities were observed at different concentrations of **1** and **5** (Figures 6C,D). These results suggest that **1** and **5** have potential as *in vitro* anti-inflammatory agents, with better or comparable activities than those of the positive control, dexamethasone (DEX).

**TABLE 3:** Inhibitory activity against LPS-induced NO production of **1**–**12**.

No.	IC_50_ (*µ*M)	No.	IC_50_ (*µ*M)
	NO production		NO production
**1**	**1.49 ± 0.31**	**7**	>40
**2**	>40	**8**	>40
**3**	>40	**9**	>40
**4**	>40	**10**	>40
**5**	**3.41 ± 0.85**	**11**	>40
**6**	>40	**12**	>40
DEX^a^	6.68 ± 0.58	—	—

a DEX was used as the positive control.

### One and Five Exerted Potent Anti-Inflammatory Effect by Restraining the M1 Macrophage Polarization Induced by LPS

**FIGURE 7:**
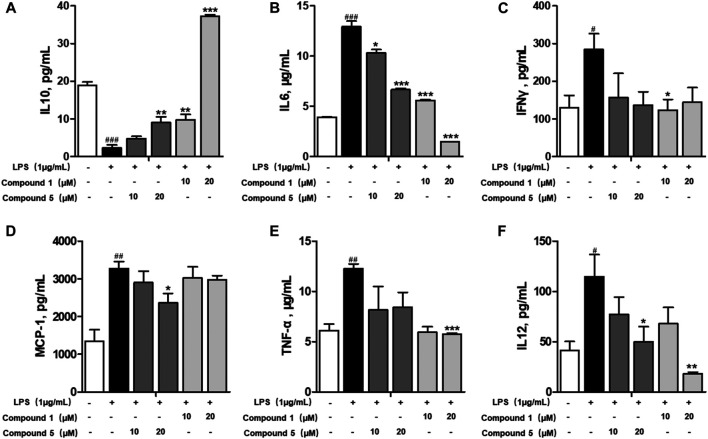
Effects of **1** and **5** on the production of cytokines by LPS-induced RAW264.7 cells. The level of IL10 **(A)**, IL6 **(B)**, IFNγ **(C)**, MCP-1 **(D)**, TNF-α **(E)**, and IL12 **(F)** produced by LPS-stimulated RAW264.7 cells treated with **1** or **5**. Data were presented as the mean ± SEM (*n* = 3). ^#^ *p* < 0.05, ^##^ *p* < 0.01, ^###^ *p* < 0.001 vs. control, *p < 0.05, **p < 0.01, ***p < 0.001 vs. model.

**FIGURE 8:**
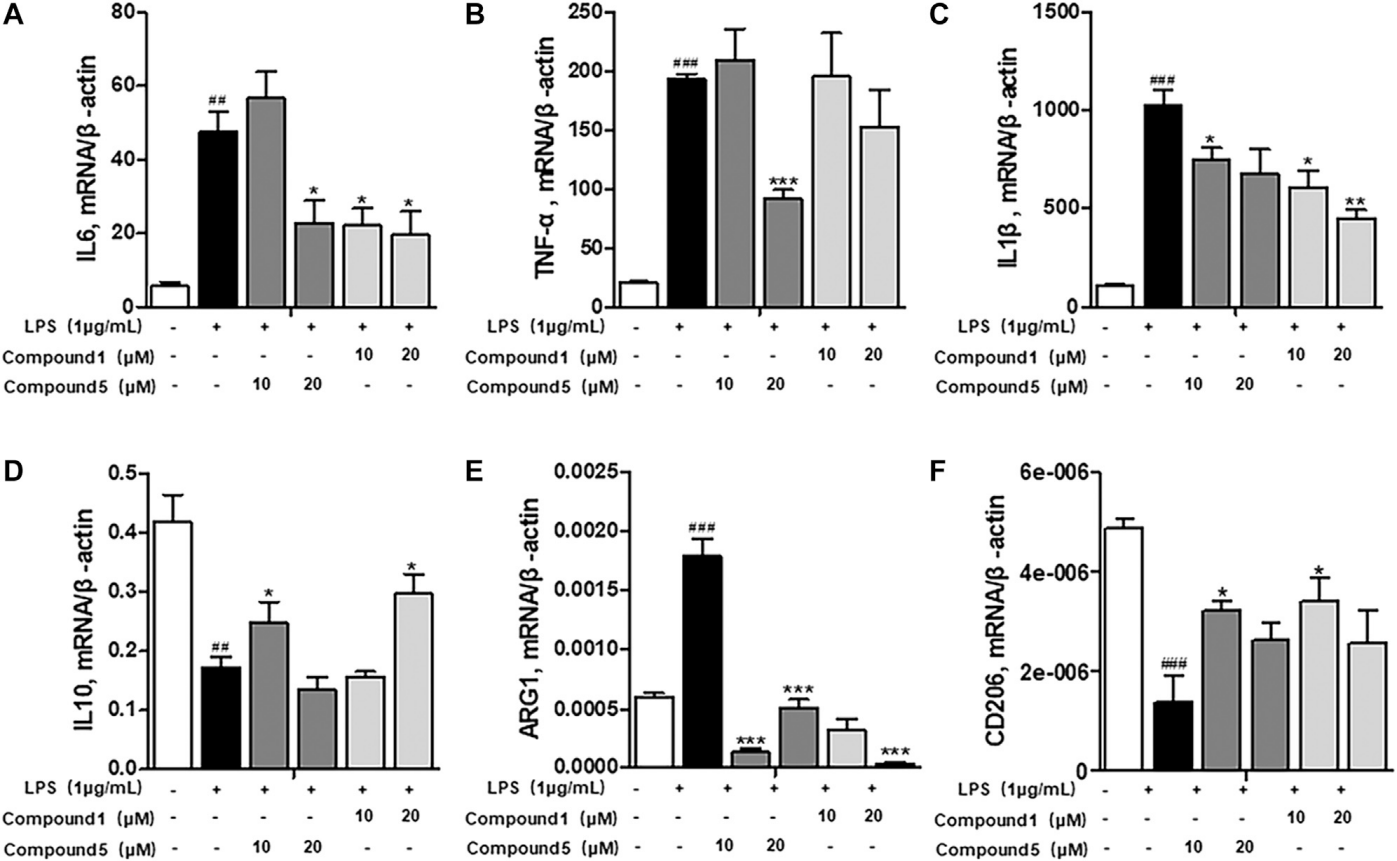
Influences of **1** and **5** on the transcription level of macrophage markers. The expression of inflammatory markers IL6 **(A)**, TNF-α **(B)**, and IL1β **(C)** and immunomodulatory markers IL10 **(D)**, ARG1 **(E)**, and CD206 **(F)** in **1-** or **5**-administrated RAW264.7 cells activated by LPS. Data were presented as the mean ± SEM (*n* = 3). ^##^ *p* < 0.01, ^###^ *p* < 0.001 vs. control, *p < 0.05, **p < 0.01, ***p < 0.001 vs. model.

Macrophages play an important role in multiple immune responses. Research studies have clarified two major macrophages, including classically activated or inflammatory (M1) and alternatively activated or anti-inflammatory (M2) macrophages (Wang et al., 2019). M1 macrophages can be stimulated by LPS, and then, they secrete high levels of pro-inflammatory cytokine factors, such as NO, COX-2, IL6, IL1β, and TNF-α. Meanwhile, M2 macrophages can be activated by IL-4 or IL-13, characterized by the high-level secretion of IL10, arginase-1 (ARG1), and the mannose receptor, C type 1 (MRC1/CD206) (Kong et al., 2019). Hence, we first detected the secretion of cytokines from the cell culture supernatant of administrated RAW264.7 cells. Our results demonstrated that **1** and **5** both elevated the level of immunoregulatory cytokine IL10 while decreasing pro-inflammatory cytokines IL6, TNF-α, IFN-γ, MCP-1, and IL12 (Figures 7A–F). These findings prompted us to conclude that both **1** and **5** can suppress the induction of M1 macrophages by LPS. As well, our qRT-PCR results exhibited that **1** and **5** significantly inhibited the transcription level of pro-inflammation M1 markers IL6, IL1β, and TNF-α (Figures 8A–D) while remarkably increasing the expression of IL10 and the anti-inflammation M2 markers ARG1 and CD206 (Figures 8E,F). Altogether, our results revealed that both **1** and **5** can have a potent anti-inflammatory effect by preventing the M1 macrophage polarization induced by LPS.

### One and Five Inhibit the Nuclear Translocation of Nuclear Factor-Kappa B p65

**FIGURE 9:**
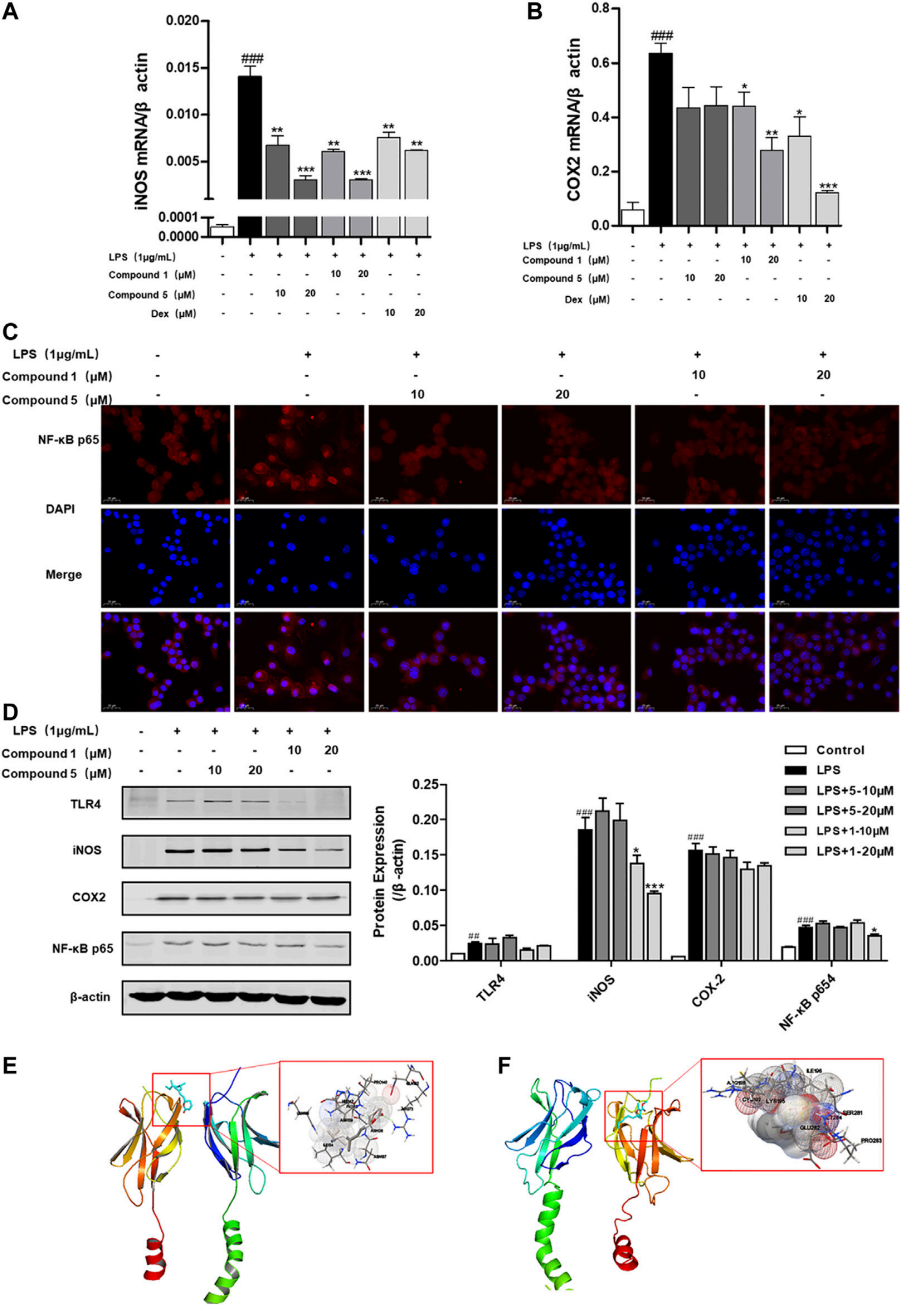
**1** and **5** inhibit the nuclear translocation of NF-κB p65 to exert anti-inflammation effect. Effects of **1** and **5** on the transcription level of inflammatory factors iNOS **(A)** and COX-2 **(B)** in LPS-induced RAW 264.7 cells. Dexamethasone (Dex) was used as a positive control. Data were presented as the mean ± SEM (*n* = 3). ^###^ *p* < 0.001 vs. control, *p < 0.05, **p < 0.01, ***p < 0.001 vs. model. **(C)** Effects of **1** and **5** on the expression of NF-κB p65 in LPS-treated RAW264.7 cells by immunofluorescence (scale bar = 20 μm). **(D)** Protein expression of TLR4, iNOS, COX-2, and NF-κB p65 in RAW264.7 cells stimulated with LPS. ^##^ *p* < 0.01, ^###^ *p* < 0.001 vs. control, *p < 0.05, **p < 0.01, ***p < 0.001 vs. model. *In silico* analysis of the interactions between NF-κB p65 and **1 (E)** and **5 (F)**, respectively.

LPS can bind to and activate cell membrane receptor toll-like receptor 4 (TLR4), thus producing a large number of NF-κB through MyD88-dependent pathways, thereby promoting M1 macrophage polarization (Ostareck and Ostareck-Lederer, 2019). We also found that both **1** and **5** can significantly depress the transcription level of inflammatory factors iNOS and COX-2 (Figures 9A,B). Meanwhile, only compound **1** can notably inhibit the protein expression of iNOS and NF-κB p65 (Figure 9D), while **5** exerts slight inhibition on the protein expression of COX-2 and NF-κB p65 (Figure 9D). NF-κB p65 is a pivotal transcriptional factor in the macrophage activation progress, which can activate and translocate into the nucleus to regulate the transcription of COX-2 and iNOS (Emam et al., 2021). We also found that both **1** and **5** can inhibit the nuclear translocation of NF-κB p65 (Figure 9C). On this basis, compounds **1** and **5** were subjected to molecular docking to NF-κB p65 (PDB ID of protein receptor: 1OY3) to gain a better understanding of the hypothetical mechanism. As a result, **1** can virtually bind to NF-κB p65 through hydrogen bonds to the binding sites Asn139 and Arg73, active residues shown in Figure 9E, with an affinity of −6.98 kcal/mol. In comparison, **5** binds to NF-κB p65 through hydrogen bonds to the binding sites Arg246 and Lys221, active residues shown in Figure 9F, with an affinity of −5.78 kcal/mol. Hence, we speculate that **1** can preferably bind to and inhibit NF-κB p65 to exert the anti-inflammatory effect.

## Discussion

To the best of our knowledge, there are very little research data on the secondary metabolites of *Aspergillus rugulosa*. Aiming to discover new chemical constituents and their pharmacological activities, we explored the secondary metabolites of this fungus. In conclusion, two new polyketide compounds, asperulosins A and B (**1**–**2**), and one new prenylated small molecule, asperulosin C (**3**), along with nine known compounds (**4**–**12**), were isolated and identified from the fungus *Aspergillus rugulosa*. Remarkably, compound **1** was determined as a unique linear chain compound with an *α ,β*-unsaturated monocyclic lactone structure originating from the polyketide family. Compound **2** was constructed by five continuous quaternary carbon atoms, which occur rarely in natural products. Except for compound **5**, the others were first isolated from the fungus *Aspergillus rugulosa*.

Macrophages play an important role in the immune system. They can be stimulated by LPS, and then, they secrete multiple pro-inflammatory factors, such as NO, COX-2, IL6, IL1β, and TNF-α (Duarte et al., 2014). Excessive NO can induce DNA damage, apoptosis, and oxidative stress, thereby amplifying the inflammatory response (Gu et al., 2020). Accordingly, all of the isolates were evaluated for anti-inflammatory activity against the production of NO in RAW264.7 stimulated by LPS. Among them, polyketide compounds **1** and **5** showed a significant inhibitory effect on NO production, with IC_50_ values of 1.49 ± 0.31 and 3.41 ± 0.85 *μ*M, respectively. Moreover, our results uncovered that **1** and **5** can also elevate the level of immunoregulatory cytokine IL10 while decreasing pro-inflammatory cytokines IL6, TNF-α, IFN-γ, MCP-1, and IL12. As well, **1** and **5** significantly inhibited the transcription level of pro-inflammation M1 markers IL6, IL1β, and TNF-α while remarkably increasing the expression of IL10 and the anti-inflammation M2 markers ARG1 and CD206. These results suggest that **1** and **5** could be considered as a basis for the synthesis of novel anti-inflammatory drugs.

NF-κB is a transcription factor that regulates the gene expression of inflammation, cell growth, proliferation, and immune response (Khan et al., 2020). NF-κB is composed of the p65 and p50 subunits. LPS can stimulate and activate the transcription activity of NF-κB p65 by promoting its translocation into the nucleus (Christian et al., 2016). Our results demonstrate that both **1** and **5** can obviously prevent the expression of NF-κB p65 in the nucleus and suppress the transcription of COX-2 and iNOS. However, **1** exerts more potent inhibition activity on the protein expression of TLR4, iNOS, and NF-κB p65 than **5**. These results are consistent with the NO inhibition IC_50_ value. Hence, we presume that **1** can suppress the expression and the nuclear translocation of NF-κB p65 to induce stronger anti-inflammatory activity than that of **5**.

In conclusion, both **1** and **5** can suppress immune response in LPS-induced RAW264.7 cells by blocking the NF-κB pathway and decreasing the production of COX-2 and iNOS. Finally, compounds **1** and **5** exhibited potential anti-inflammatory activities *in vitro* that were comparable to those of the positive control, DEX.

## Data Availability

The original contributions presented in the study are included in the article/Supplementary Material; further inquiries can be directed to the corresponding authors.

## References

[B1] Almassi F. Ghisalberti E. L. Narbey M. J. Sivasithamparam K. (1991). New Antibiotics from Strains of Trichoderma harzianum. J. Nat. Prod. 54, 396–402. 10.1021/np50074a008

[B2] Alvarez-Suarez J. M. Carrillo-Perdomo E. Aller A. Giampieri F. Gasparrini M. González-Pérez L. (2017). Anti-inflammatory Effect of Capuli Cherry against LPS-Induced Cytotoxic Damage in RAW 264.7 Macrophages. Food Chem. Toxicol. 102, 46–52. 10.1016/j.fct.2017.01.024 28137607

[B3] Ballantine J. A. Ferrito V. Hassall C. H. Jones V. I. P. (1969). Aspertetronin A and B, Two Novel Tetronic Acid Derivatives Produced by a Blocked Mutant of Aspergillus rugulosus. J. Chem. Soc. C, 56–61. 10.1039/J39690000056

[B4] Bifulco G. Dambruoso P. Gomez-Paloma L. Riccio R. (2007). Determination of Relative Configuration in Organic Compounds by NMR Spectroscopy and Computational Methods. Chem. Rev. 107, 3744–3779. 10.1021/cr030733c 17649982

[B5] Burghart-Stoll H. Brückner R. (2012). Total Syntheses of the Gregatins A-D and Aspertetronin A: Structure Revisions of These Compounds and of Aspertetronin B, Together with Plausible Structure Revisions of Gregatin E, Cyclogregatin, Graminin A, the Penicilliols A and B, and the Huaspenones A. Eur. J. Org. Chem. 2012, 3978–4017. 10.1002/ejoc.201200207

[B6] Cacho R. A. Jiang W. Chooi Y.-H. Walsh C. T. Tang Y. (2012). Identification and Characterization of the Echinocandin B Biosynthetic Gene Cluster from Emericella rugulosa NRRL 11440. J. Am. Chem. Soc. 134, 16781–16790. 10.1021/ja307220z 22998630 PMC3482383

[B7] Choudhary M. I. Musharraf S. G. Mukhmoor T. Shaheen F. Ali S. Rahman A.-u. (2004). Isolation of Bioactive Compounds from Aspergillus terreus. Naturforsch. B 59, 324–328. 10.1515/znb-2004-0315

[B8] Christian F. Smith E. Carmody R. (2016). The Regulation of NF-Κb Subunits by Phosphorylation. Cells 5, 12. 10.3390/cells5010012 PMC4810097 26999213

[B9] Duarte J. Francisco V. Perez-Vizcaino F. (2014). Modulation of Nitric Oxide by Flavonoids. Food Funct. 5, 1653–1668. 10.1039/c4fo00144c 24901042

[B10] Elsebai M. F. Ghabbour H. A. Legrave N. Fontaine-Vive F. Mehiri M. (2018). New Bioactive Chlorinated Cyclopentene Derivatives from the marine-derived Fungus Phoma Sp. Med. Chem. Res. 27, 1885–1892. 10.1007/s00044-018-2201-1

[B11] Emam S. H. Sonousi A. Osman E. O. Hwang D. Kim G.-D. Hassan R. A. (2021). Design and Synthesis of Methoxyphenyl- and Coumarin-Based Chalcone Derivatives as Anti-inflammatory Agents by Inhibition of NO Production and Down-Regulation of NF-Κb in LPS-Induced RAW264.7 Macrophage Cells. Bioorg. Chem. 107, 104630. 10.1016/j.bioorg.2021.104630 33476864

[B12] González-Medina M. Owen J. R. El-Elimat T. Pearce C. J. Oberlies N. H. Figueroa M. (2017). Scaffold Diversity of Fungal Metabolites. Front. Pharmacol. 8, 180. 10.3389/fphar.2017.00180 28420994 PMC5376591

[B13] Gu I. Brownmiller C. Stebbins N. B. Mauromoustakos A. Howard L. Lee S.-O. (2020). Berry Phenolic and Volatile Extracts Inhibit Pro-inflammatory Cytokine Secretion in LPS-Stimulated RAW264.7 Cells through Suppression of NF-Κb Signaling Pathway. Antioxidants 9, 871. 10.3390/antiox9090871 PMC7554842 32942640

[B14] Herkommer D. Schmalzbauer B. Menche D. (2014). Sequential Catalysis for Stereoselective Synthesis of Complex Polyketides. Nat. Prod. Rep. 31, 456–467. 10.1039/C3NP70093C 24362363

[B15] Hertweck C. (2009). The Biosynthetic Logic of Polyketide Diversity. Angew. Chem. Int. Ed. 48, 4688–4716. 10.1002/anie.200806121 19514004

[B16] Hu Z. Ye Y. Zhang Y. (2021). Large-scale Culture as a Complementary and Practical Method for Discovering Natural Products with Novel Skeletons. Nat. Prod. Rep. 10.1039/D0NP00069H 33650608

[B17] Islam M. S. Ishigami K. Watanabe H. (2007). Synthesis of (−)-mellein, (+)-ramulosin, and Related Natural Products. Tetrahedron. 63, 1074–1079. 10.1016/j.tet.2006.11.068

[B18] Khan H. Ullah H. Castilho P. C. M. F. Gomila A. S. D'Onofrio G. Filosa R. (2020). Targeting NF-Κb Signaling Pathway in Cancer by Dietary Polyphenols. Crit. Rev. Food Sci. Nutr. 60, 2790–2800. 10.1080/10408398.2019.1661827 31512490

[B19] Kwok O. C. H. Plattner R. Weisleder D. Wicklow D. T. (1992). A Nematicidal Toxin fromPleurotus Ostreatus NRRL 3526. J. Chem. Ecol. 18, 127–136. 10.1007/BF00993748 24254904

[B20] Lacoske M. H. Theodorakis E. A. (2015). Spirotetronate Polyketides as Leads in Drug Discovery. J. Nat. Prod. 78, 562–575. 10.1021/np500757w 25434976 PMC4380204

[B21] Lee W.-S. Shin J.-S. Jang D. S. Lee K.-T. (2016). Cnidilide, an Alkylphthalide Isolated from the Roots of Cnidium Officinale, Suppresses LPS-Induced NO, PGE 2, IL-1β, IL-6 and TNF-α Production by AP-1 and NF-Κb Inactivation in RAW 264.7 Macrophages. Int. Immunopharmacology 40, 146–155. 10.1016/j.intimp.2016.08.021 27591413

[B22] Li H. Feng W. Li X. Kang X. Yan S. Chao M. (2020). Terreuspyridine: An Unexpected Pyridine-Fused Meroterpenoid Alkaloid with a Tetracyclic 6/6/6/6 Skeleton from Aspergillus terreus. Org. Lett. 22, 7041–7046. 10.1021/acs.orglett.0c02641 32841036

[B23] Medzhitov R. (2010). Inflammation 2010: New Adventures of an Old Flame. Cell 140, 771–776. 10.1016/j.cell.2010.03.006 20303867

[B24] Newman D. J. Cragg G. M. (2020). Natural Products as Sources of New Drugs over the Nearly Four Decades from 01/1981 to 09/2019. J. Nat. Prod. 83, 770–803. 10.1021/acs.jnatprod.9b01285 32162523

[B25] Okamoto T. Sanda T. Asamitsu K. (2007). NF-κB Signaling and Carcinogenesis. Cpd, 13, 447–462. 10.2174/138161207780162944 17348842

[B26] Ostareck D. H. Ostareck-Lederer A. (2019). RNA-binding Proteins in the Control of LPS-Induced Macrophage Response. Front. Genet. 10. 10.3389/fgene.2019.00031 PMC6369361 30778370

[B27] Qi C. Bao J. Wang J. Zhu H. Xue Y. Wang X. (2016). Asperterpenes A and B, Two Unprecedented Meroterpenoids from Aspergillus terreus with BACE1 Inhibitory Activities. Chem. Sci. 7, 6563–6572. 10.1039/C6SC02464E 28042460 PMC5131395

[B28] Qiao Y. Xu Q. Feng W. Tao L. Li X.-N. Liu J. (2019). Asperpyridone A: An Unusual Pyridone Alkaloid Exerts Hypoglycemic Activity through the Insulin Signaling Pathway. J. Nat. Prod. 82 (10), 2925–2930. 10.1021/acs.jnatprod.9b00188 31490677

[B29] Qiao Y. Xu Q. Hu Z. Li X.-N. Xiang M. Liu J. (2016). Diterpenoids of the Cassane Type from caesalpinia Decapetala. J. Nat. Prod. 79 (12), 3134–3142. 10.1021/acs.jnatprod.6b00910 27966950

[B30] Van Wagoner R. M. Satake M. Wright J. L. C. (2014). Polyketide Biosynthesis in Dinoflagellates: what Makes it Different?. Nat. Prod. Rep. 31, 1101–1137. 10.1039/C4NP00016A 24930430

[B31] Vu M. Herfindal L. Juvik O. J. Vedeler A. Haavik S. Fossen T. (2016). Toxic Aromatic Compounds from Fruits of Narthecium Ossifragum L. Phytochemistry 132, 76–85. 10.1016/j.phytochem.2016.09.010 27720435

[B32] Wang Y. Smith W. Hao D. He B. Kong L. (2019). M1 and M2 Macrophage Polarization and Potentially Therapeutic Naturally Occurring Compounds. Int. Immunopharmacology 70, 459–466. 10.1016/j.intimp.2019.02.050 30861466

[B33] Zhang W. Lou H.-X. Li G.-Y. Wu H.-M. (2003). A New Triterpenoid fromEntodon Okamuraebroth. J. Asian Nat. Prod. Res. 5, 189–195. 10.1080/1028602031000082016 12931851

[B34] Zhu H. Chen C. Tong Q. Li X.-N. Yang J. Xue Y. (2016). Epicochalasines A and B: Two Bioactive Merocytochalasans Bearing Caged Epicoccine Dimer Units from Aspergillus flavipes. Angew. Chem. Int. Ed. 55, 3486–3490. 10.1002/anie.201511315 26836964

[B35] Zhu H. Chen C. Tong Q. Yang J. Wei G. Xue Y. (2017). Asperflavipine A: A Cytochalasan Heterotetramer Uniquely Defined by a Highly Complex Tetradecacyclic Ring System from Aspergillus flavipes QCS12. Angew. Chem. Int. Ed. 56, 5242–5246. 10.1002/anie.201701125 28378450

