# Supplementary material for: New Polyketides With Anti-Inflammatory Activity From the Fungus Aspergillus rugulosa
Source: Front Pharmacol. 2021 Jun 21;12:700573. doi: 10.3389/fphar.2021.700573 (PMC8256160; doi:10.3389/fphar.2021.700573)
Supplement: Supplementary file 1 [file DataSheet1.pdf]

New Polyketides with Anti-Inflammatory Activity from the Fungus *Aspergillus Rugulosa*

Qianqian Xu,[1,†] Yuben Qiao,[1,†] Zijun Zhang,[1,†] Yanfang Deng,[1] Tianqi Chen,[1] Li Tao,[3] Qiaoxin Xu,[1] Junjun Liu,[1] Weiguang Sun,[1] Ying Ye,[1] Yuanyuan Lu,[2,*] Changxing Qi,[1,*] and Yonghui Zhang[1,*]

[1] Hubei Key Laboratory of Natural Medicinal Chemistry and Resource Evaluation, School of Pharmacy, Tongji Medical College, Huazhong University of Science and Technology, Wuhan 430030, People's Republic of China

[2] Maternal and Child Health Hospital of Hubei Province, Tongji Medical College, Huazhong University of Science and Technology, People's Republic of China

[3] Ezhou Central Hospital, Ezhou 436000, People's Republic of China

*Corresponding author Tel.: (86) 27-83692892
E-mail: zhangyh@mails.tjmu.edu.cn (Y.Z.); qichangxing@hust.edu.cn (C.Q.); and Luyuanyuan@hust.edu.cn (Y.L.).

Contents of Supporting Information

Figure S1. ^{1}H NMR spectrum of asperulosin A (**1**) in $CDCl_3$. 3
Figure S2. ^{13}C NMR and DEPT spectra of asperulosin A (**1**) in $CDCl_3$. 4
Figure S3. HSQC spectrum of asperulosin A (**1**) in $CDCl_3$. 5
Figure S4. HMBC spectrum of asperulosin A (**1**) in $CDCl_3$. 6
Figure S5. ^{1}H–^{1}H COSY spectrum of asperulosin A (**1**) in $CDCl_3$. 7
Figure S7. HRESIMS spectrum of asperulosin A (**1**). 9
Figure S8. UV spectrum of asperulosin A (**1**). 9
Figure S9. IR spectrum of asperulosin A (**1**). 10
Figure S10. ^{1}H NMR spectrum of asperulosin B (**2**) in MeOH-d_4. 11
Figure S11. ^{13}C NMR and DEPT spectra of asperulosin B (**2**) in MeOH-d_4. 12
Figure S12. HSQC spectrum of asperulosin B (**2**) in MeOH-d_4. 13
Figure S13. HMBC spectrum of asperulosin B (**2**) in MeOH-d_4. 14
Figure S14. HRESIMS spectrum of asperulosin B (**2**). 15
Figure S15. IR spectrum of asperulosin B (**2**). 16
Figure S16. ^{1}H NMR spectrum of asperulosin C (**3**) in $CDCl_3$. 17
Figure S17. ^{13}C NMR and DEPT spectra of asperulosin C (**3**) in $CDCl_3$. 18
Figure S18. HSQC spectrum of asperulosin C (**3**) in $CDCl_3$. 19
Figure S19. HMBC spectrum of asperulosin C (**3**) in $CDCl_3$. 20
Figure S20. ^{1}H–^{1}H COSY spectrum of asperulosin C (**3**) in $CDCl_3$. 21
Figure S21. HRESIMS spectrum of asperulosin C (**3**). 22
Figure S22. IR spectrum of asperulosin C (**3**). 23
Figure S23. UV spectrum of asperulosin C (**3**). 24
Table S1. The corresponding primer sequences used in the experiment. 25

Figure S1. ^{1}H NMR spectrum of asperulosin A (**1**) in $CDCl_3$**.**

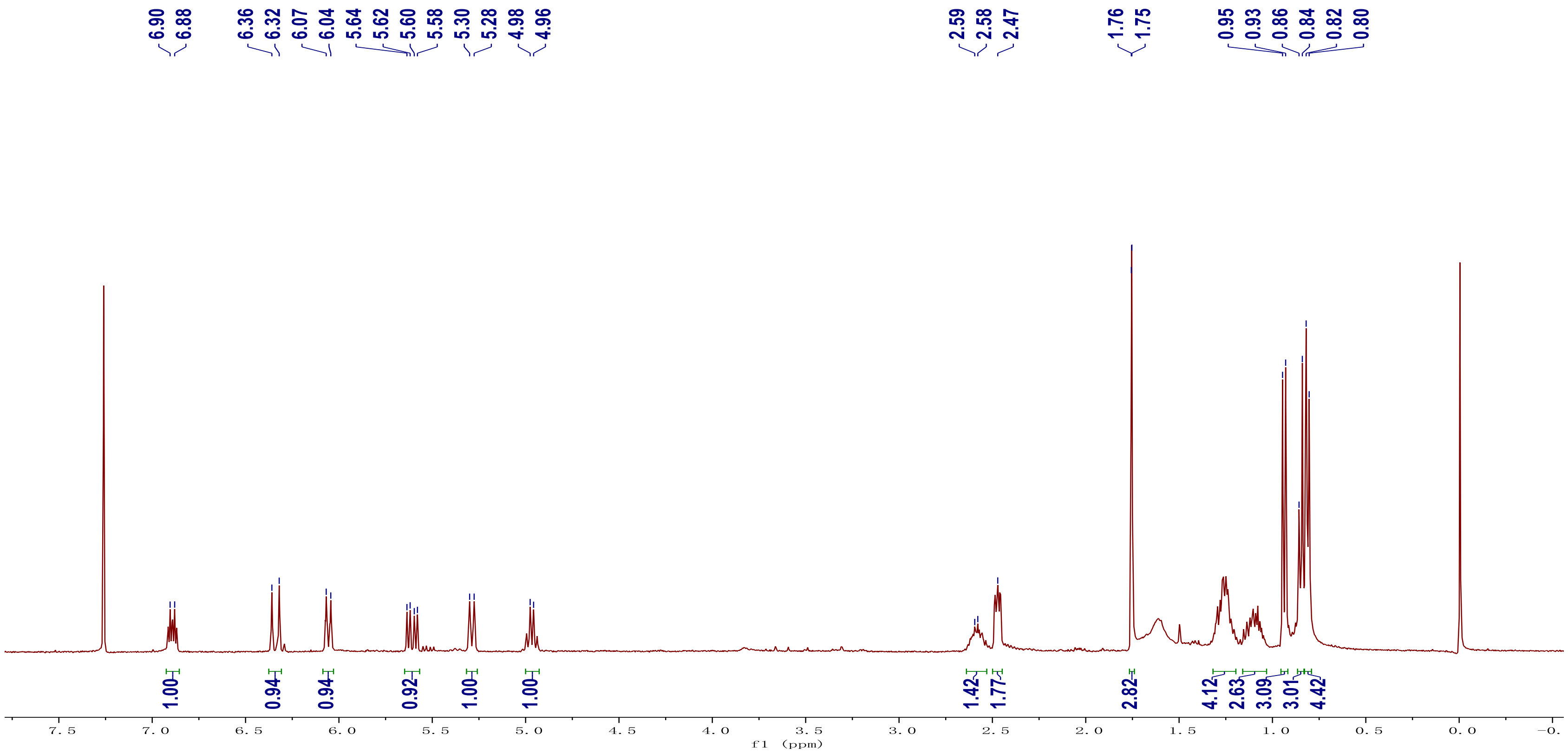

Figure S2. ^{13}C NMR and DEPT spectra of asperulosin **A** (1) in $CDCl_3$.

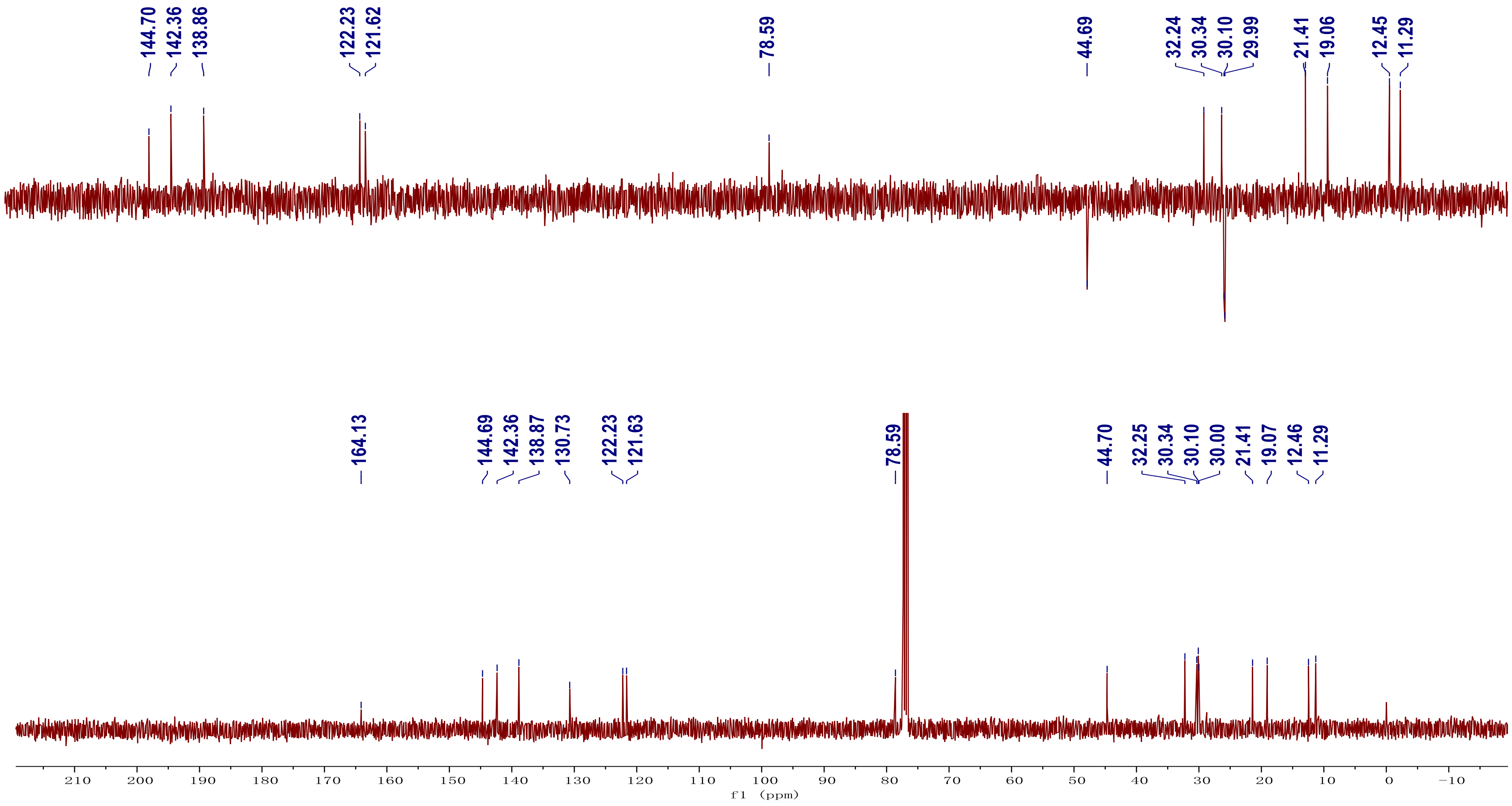

Figure S3. HSQC spectrum of asperulosin A (**1**) in $CDCl_3$.

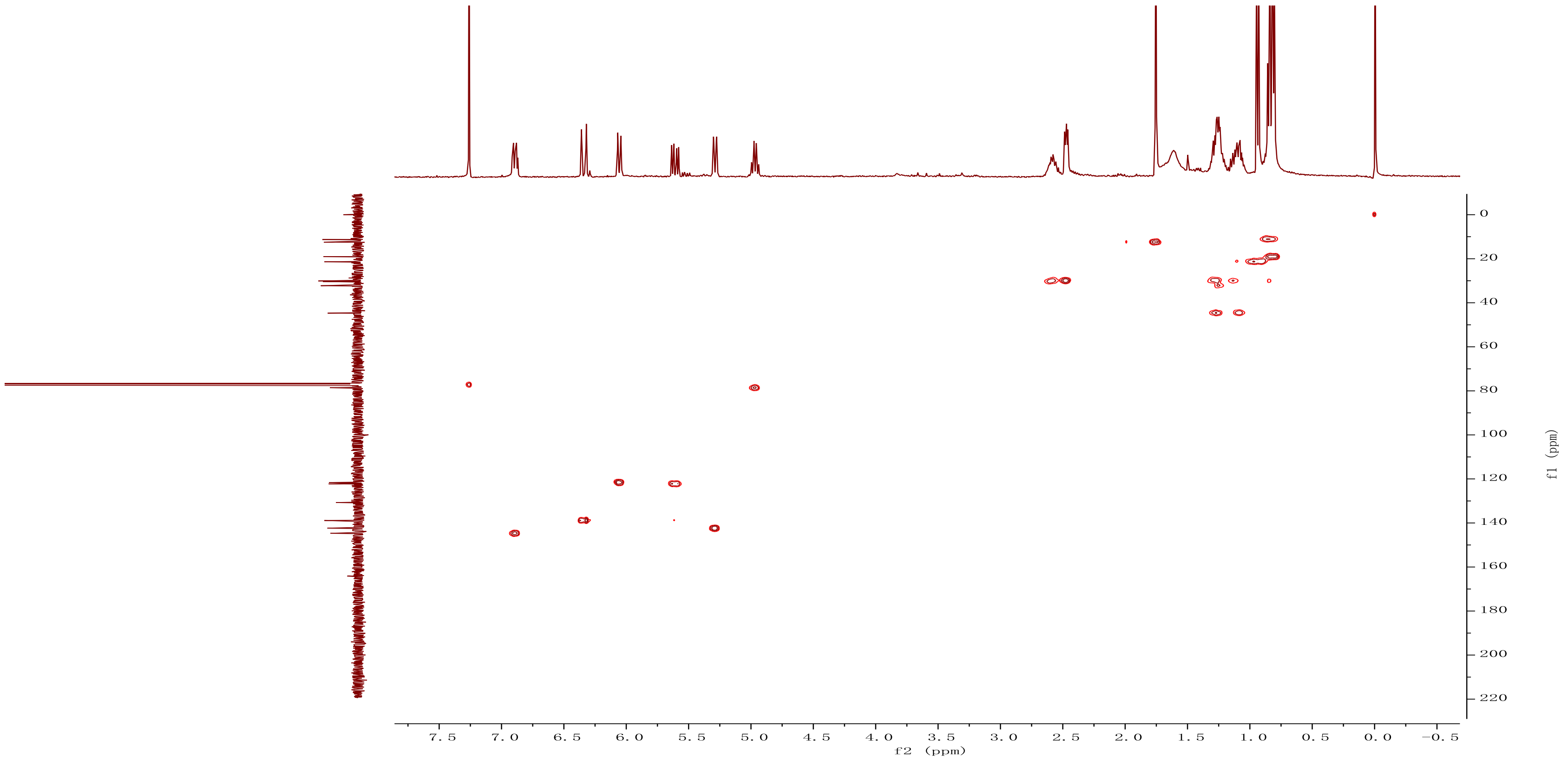

Figure S4. HMBC spectrum of asperulosin A (1) in $CDCl_3$.

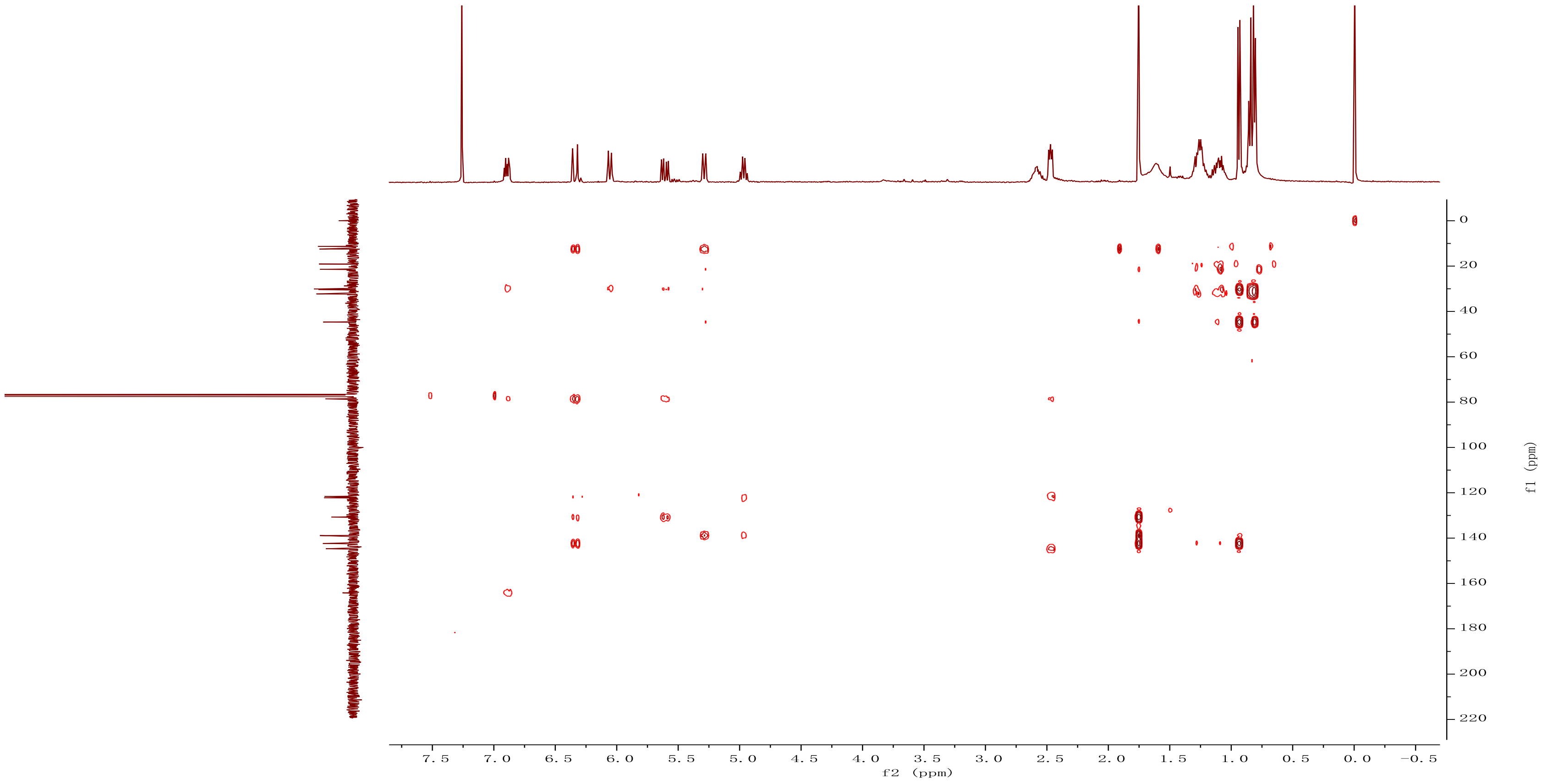

Figure S5. ^{1}H–^{1}H COSY spectrum of asperulosin A (1) in $CDCl_3$.

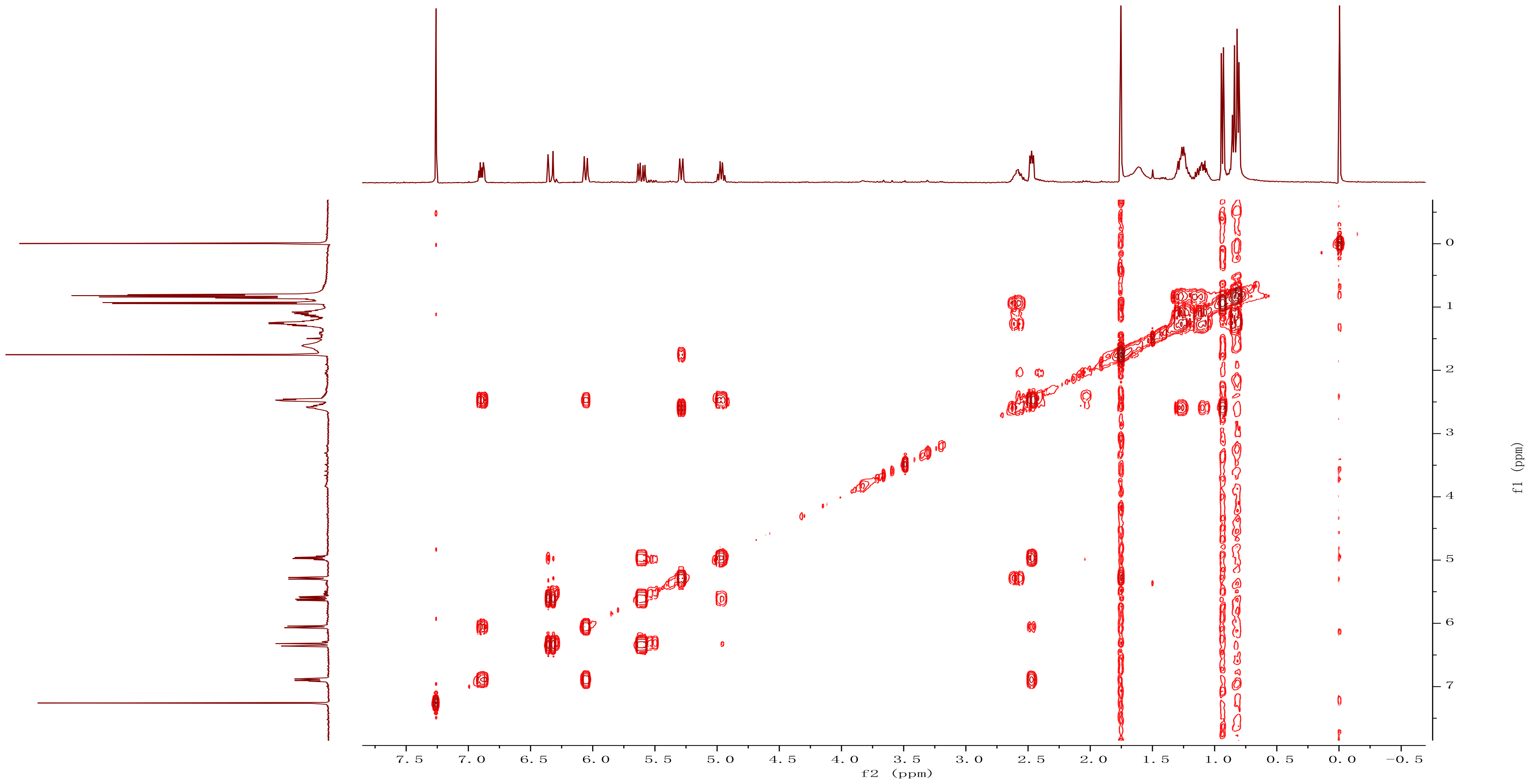

Figure S6. NOESY spectrum of asperulosin A (**1**) in $CDCl_3$.

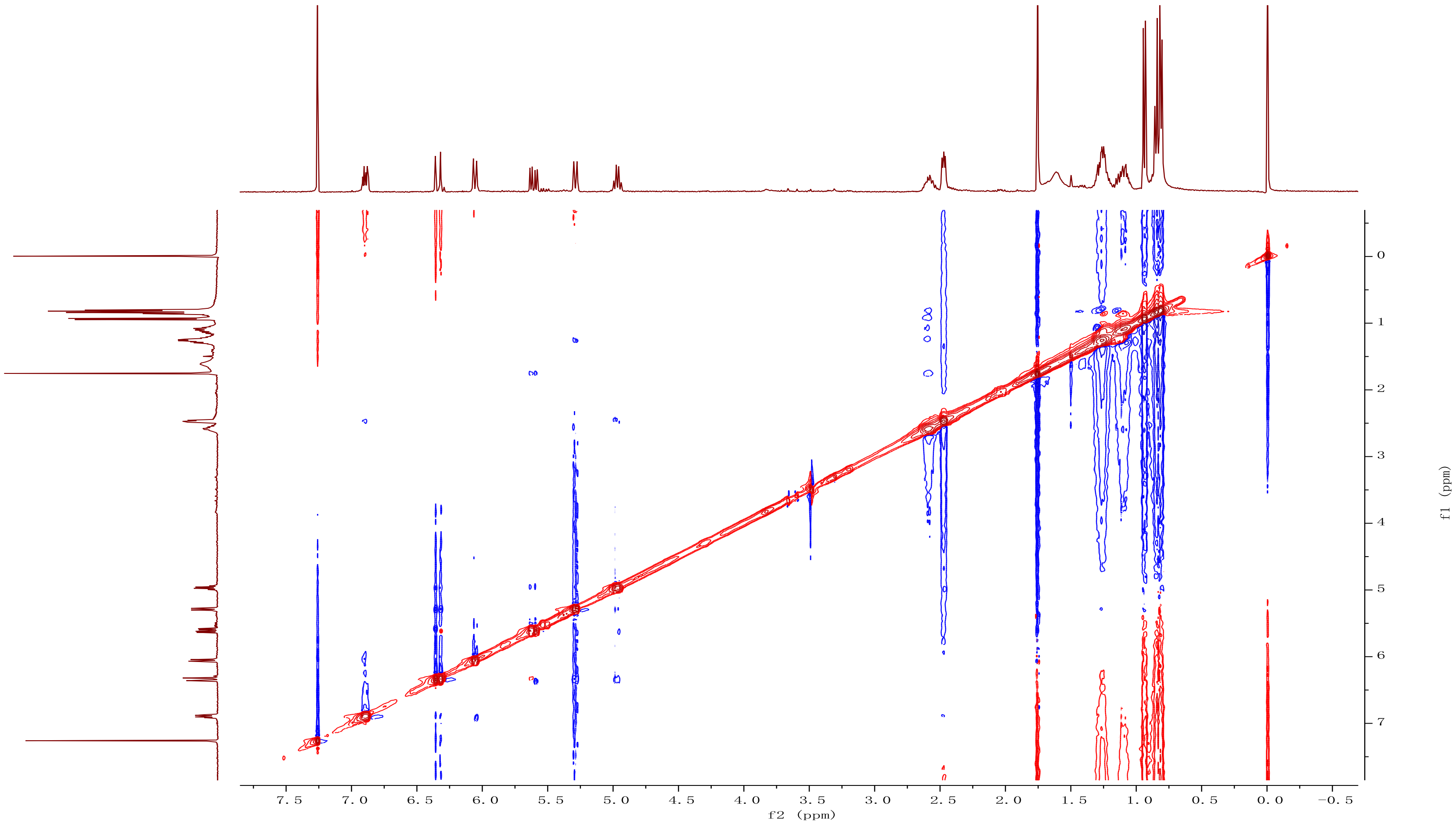

Figure S7. HRESIMS spectrum of asperulosin A (**1**).

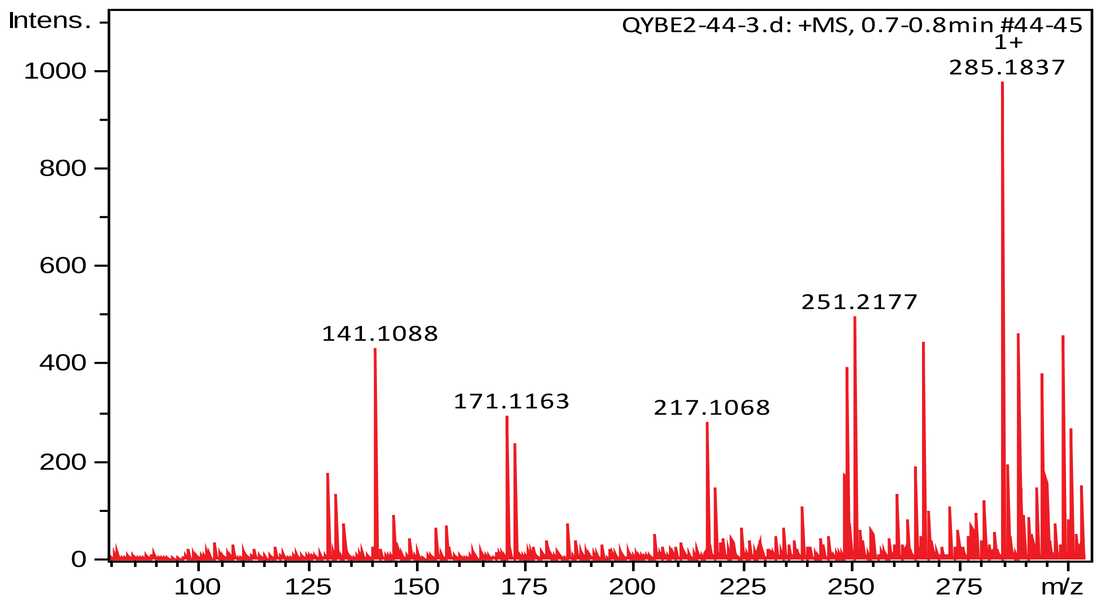

Figure S8. UV spectrum of asperulosin A (**1**).

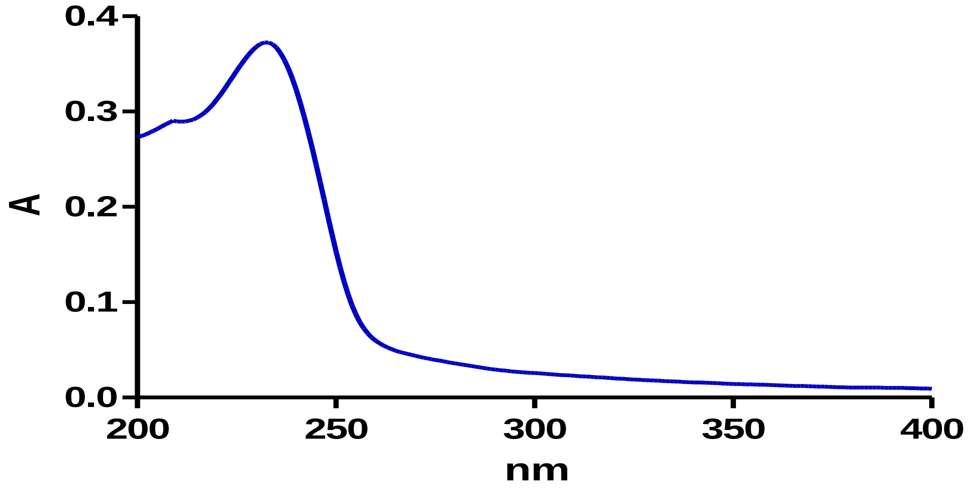

Figure S9. IR spectrum of asperulosin A (1).

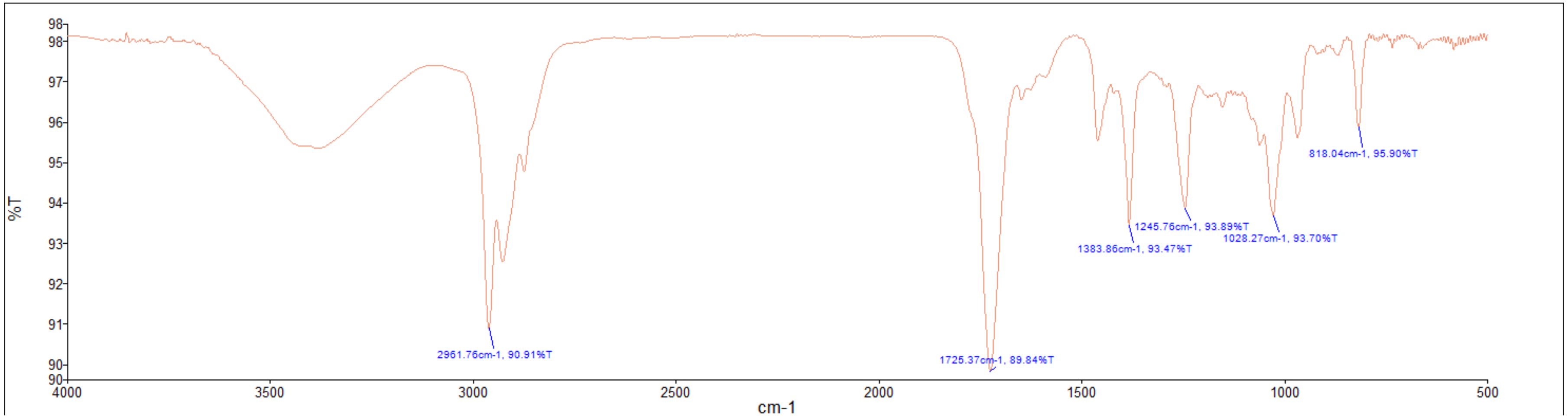

Figure S10. ^{1}H NMR spectrum of asperulosin B (**2**) in MeOH-d_4**.**

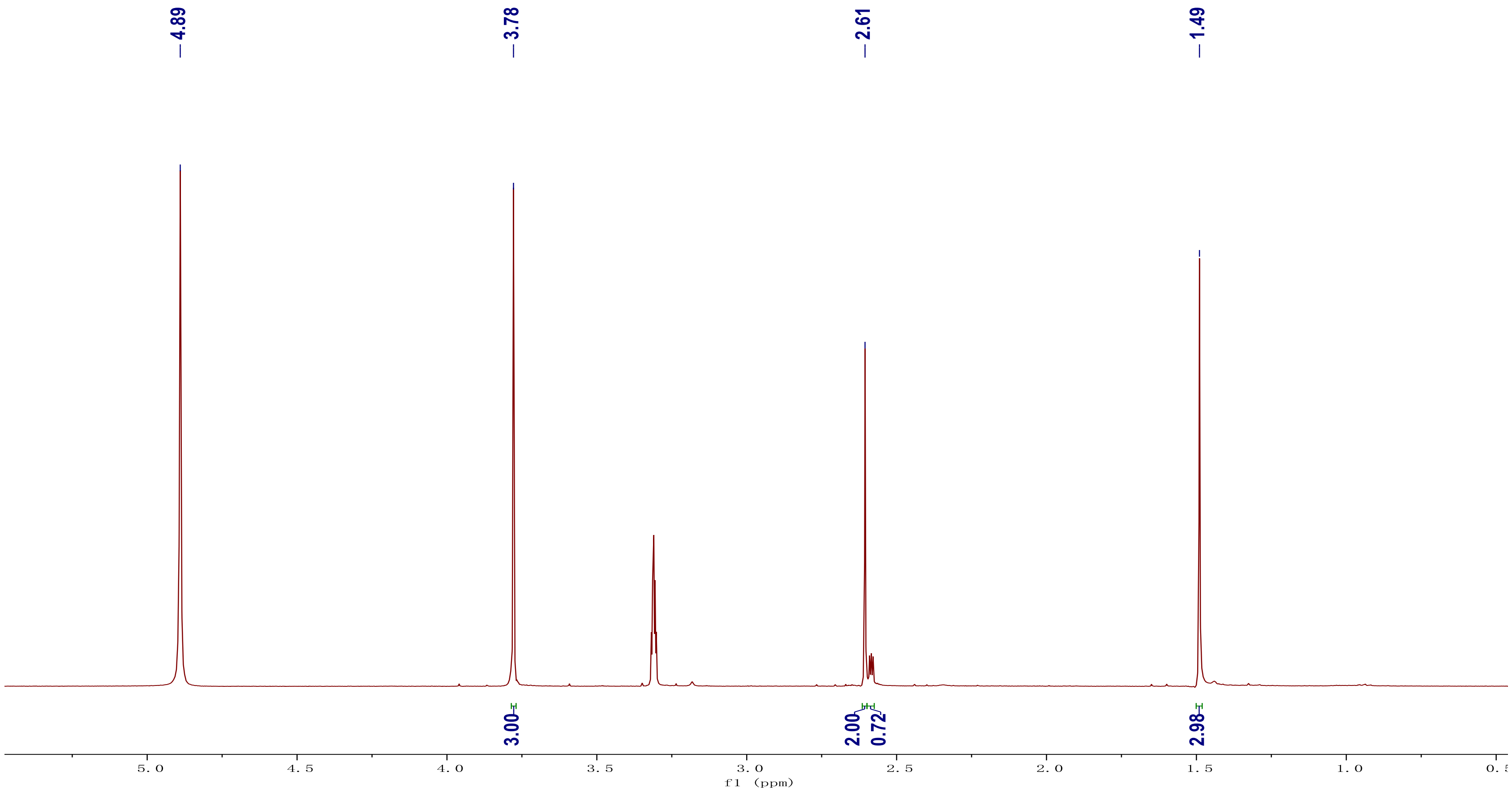

Figure S11. ^{13}C NMR and DEPT spectra of asperulosin B (**2**) in MeOH-d_4.

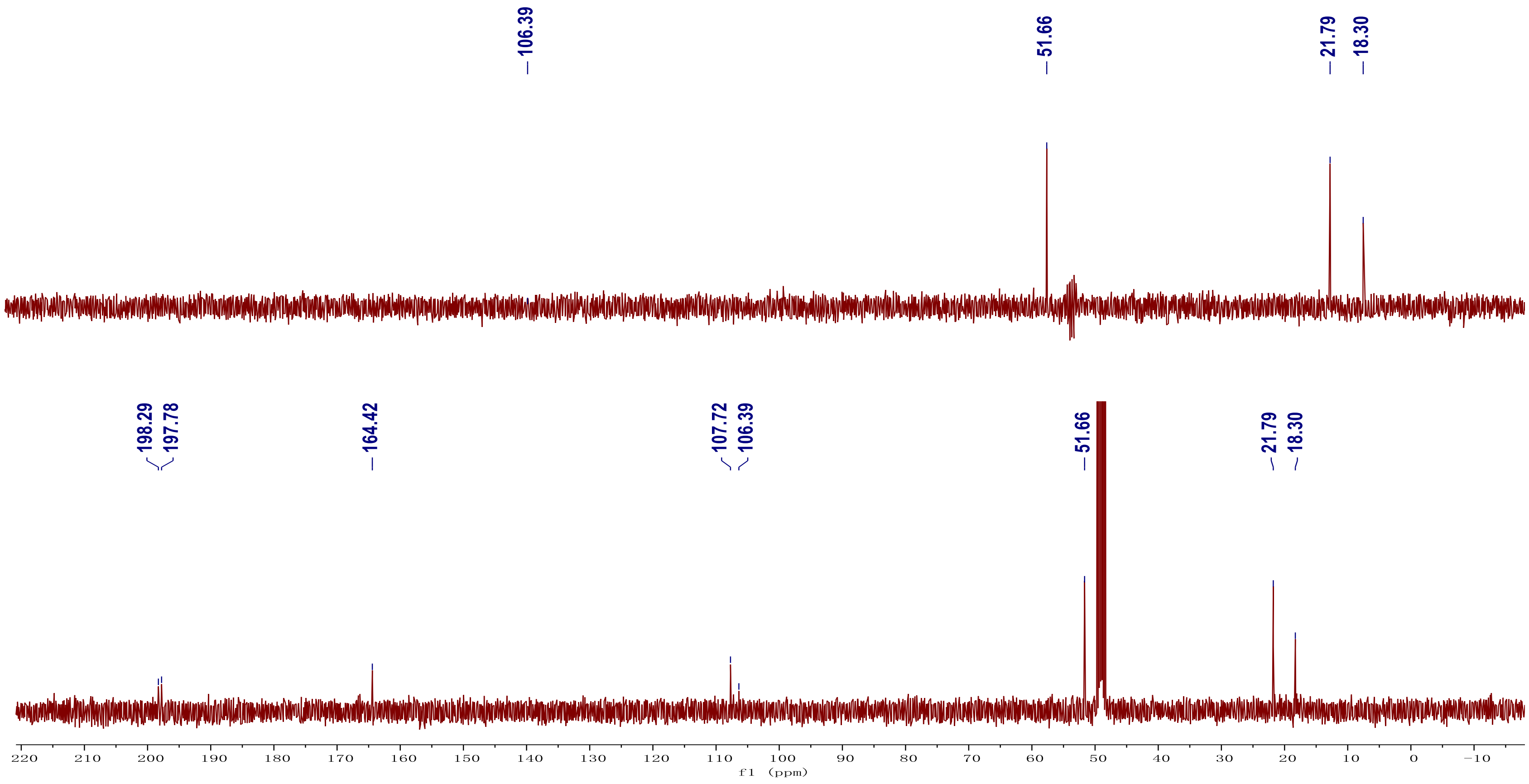

Figure S12. HSQC spectrum of asperulosin B (2) in MeOH-d_4.

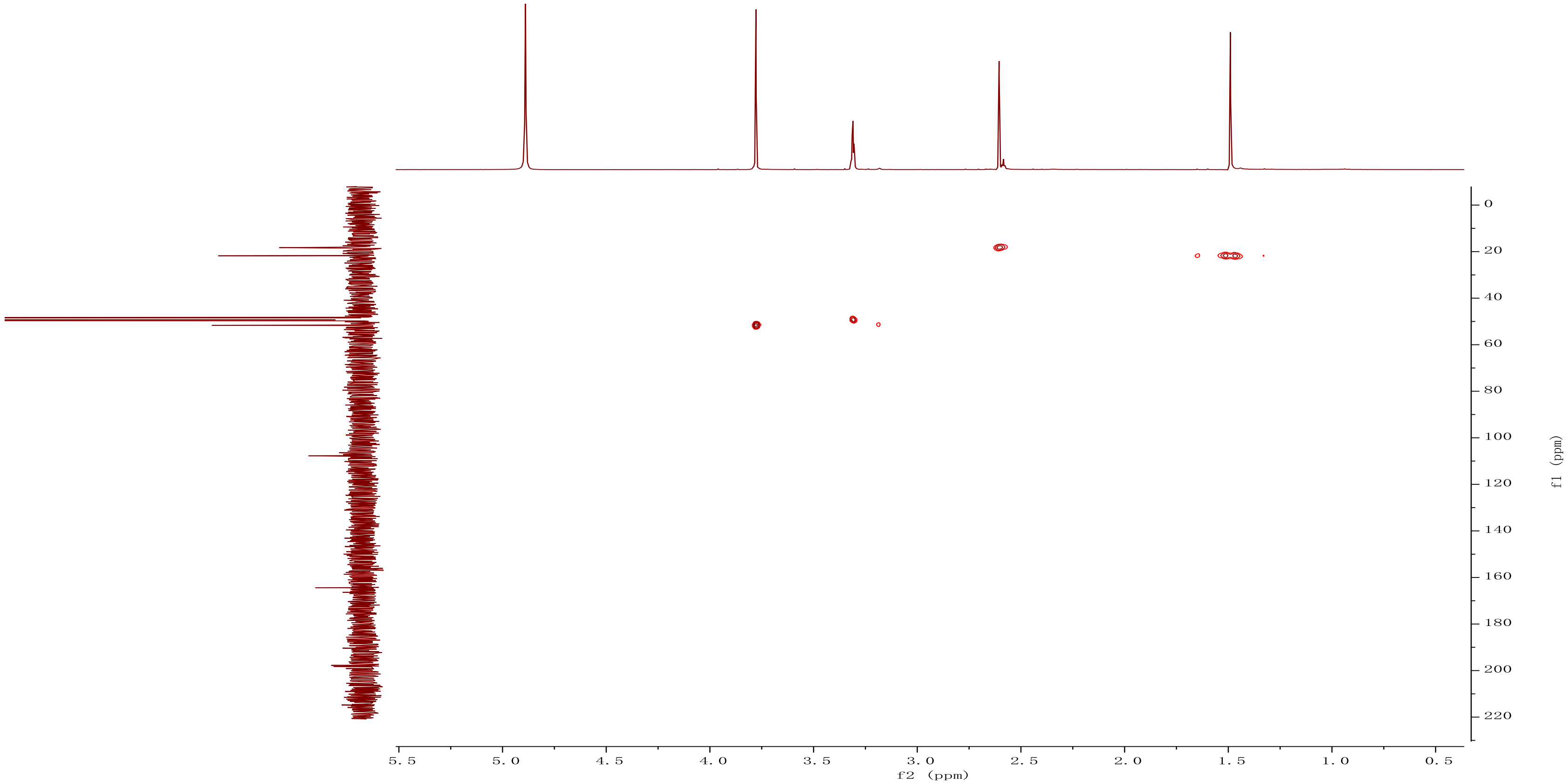

Figure S13. HMBC spectrum of asperulosin B (2) in MeOH-d_4.

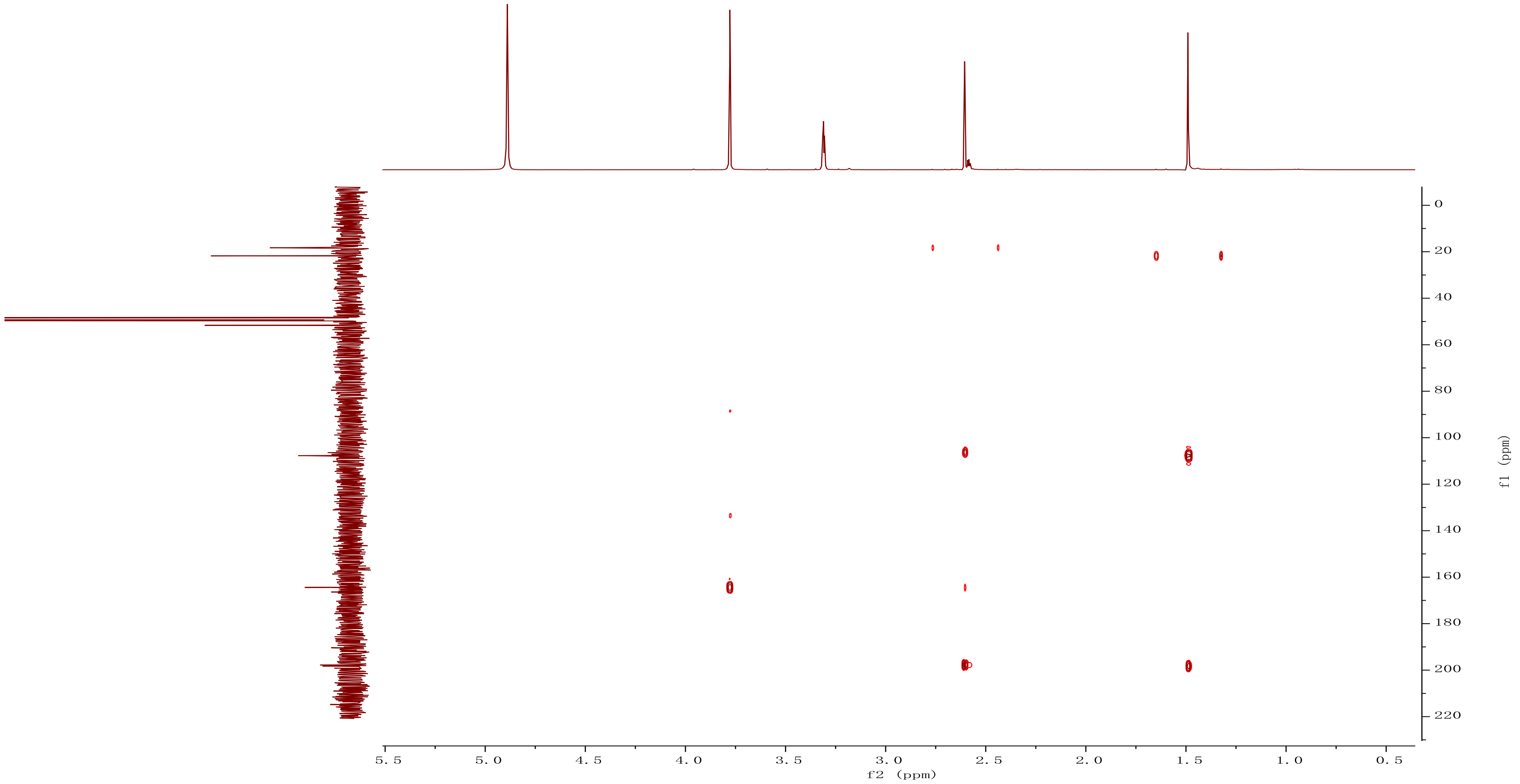

Figure S14. HRESIMS spectrum of asperulosin B (**2**).

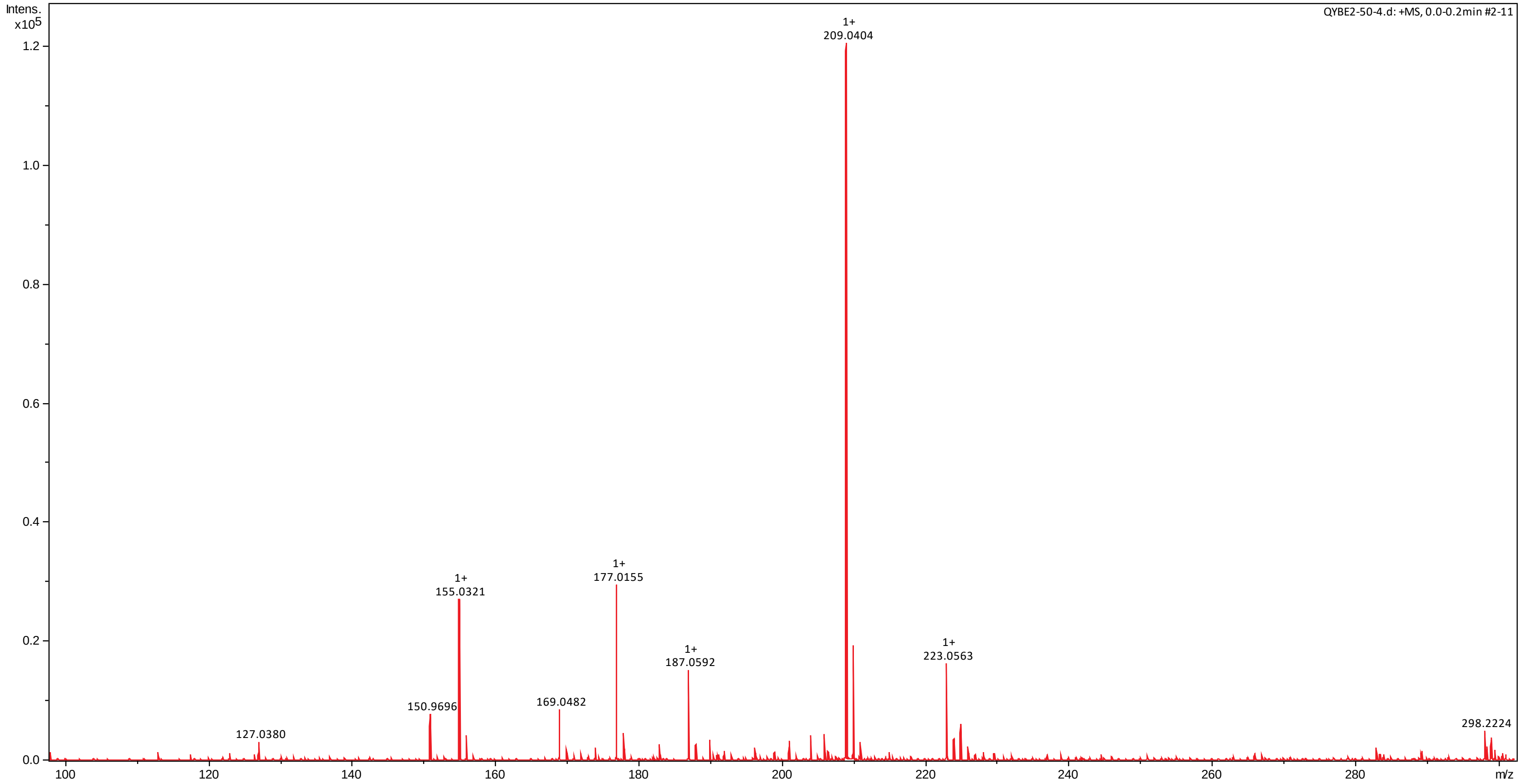

Figure S15. IR spectrum of asperulosin B (**2**).

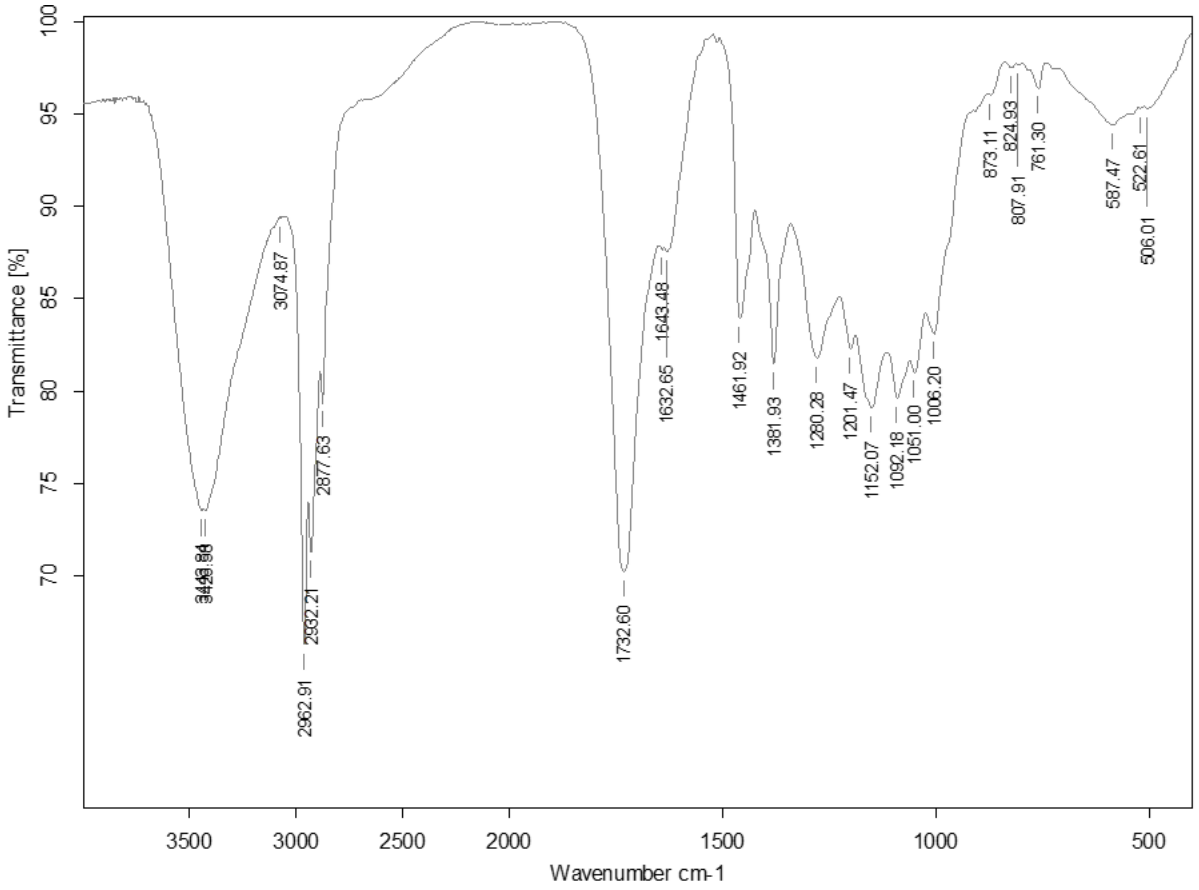

Figure S16. ^{1}H NMR spectrum of asperulosin C (**3**) in $CDCl_3$.

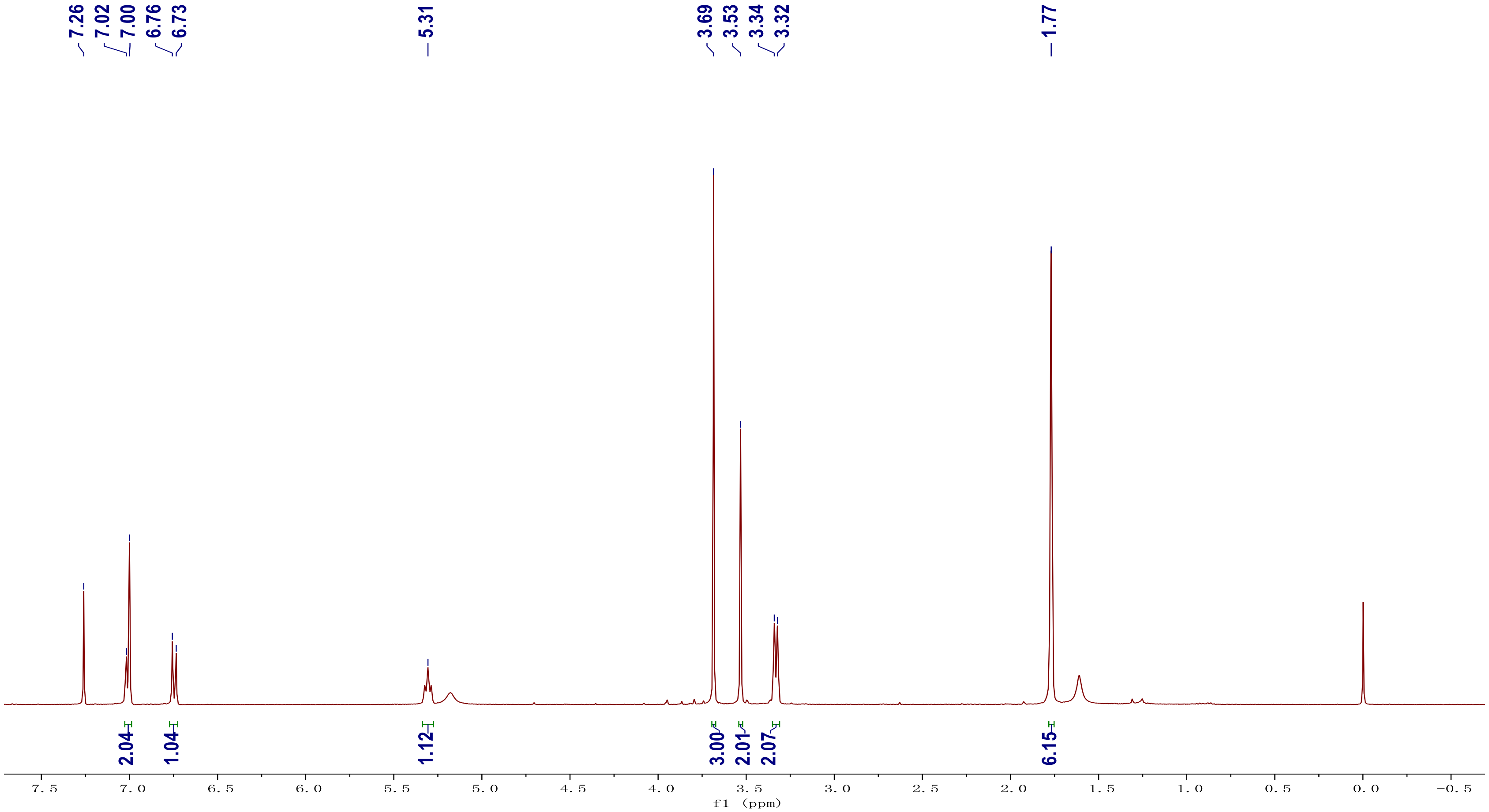

Figure S17. ^{13}C NMR and DEPT spectra of asperulosin C (**3**) in $CDCl_3$.

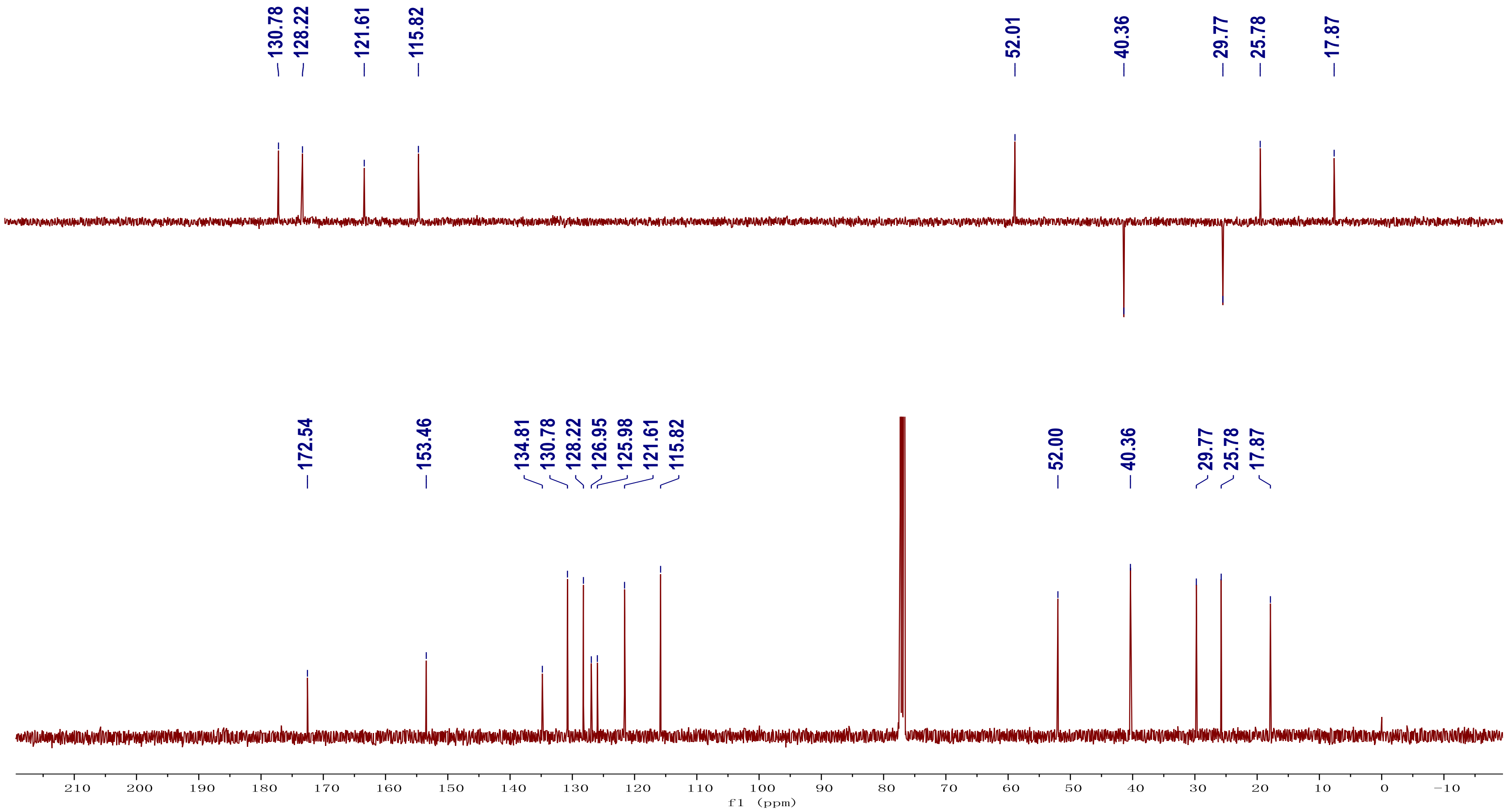

Figure S18. HSQC spectrum of asperulosin C (3) in CDCl3.

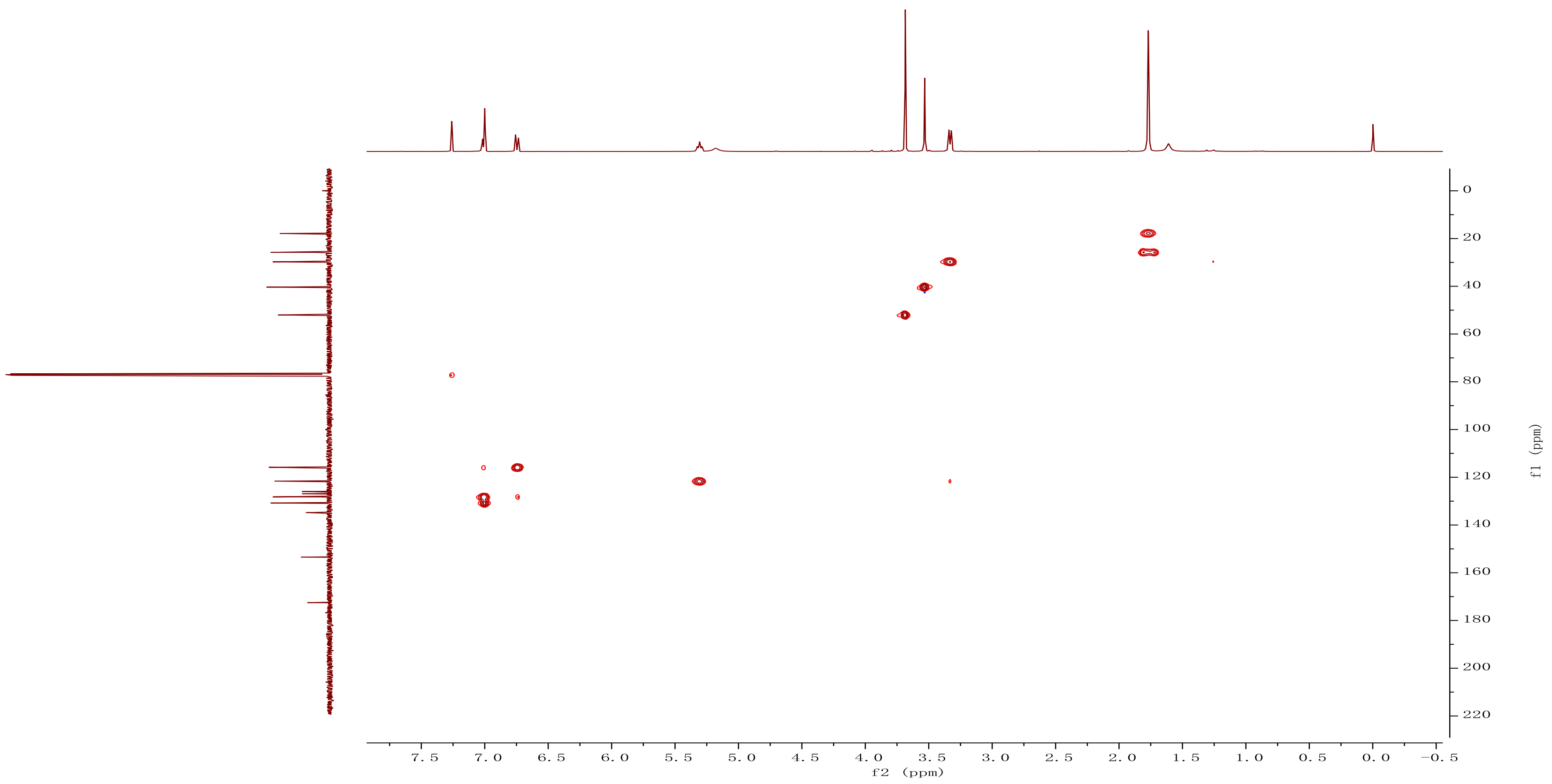

Figure S19. HMBC spectrum of asperulosin C (**3**) in $CDCl_3$**.**

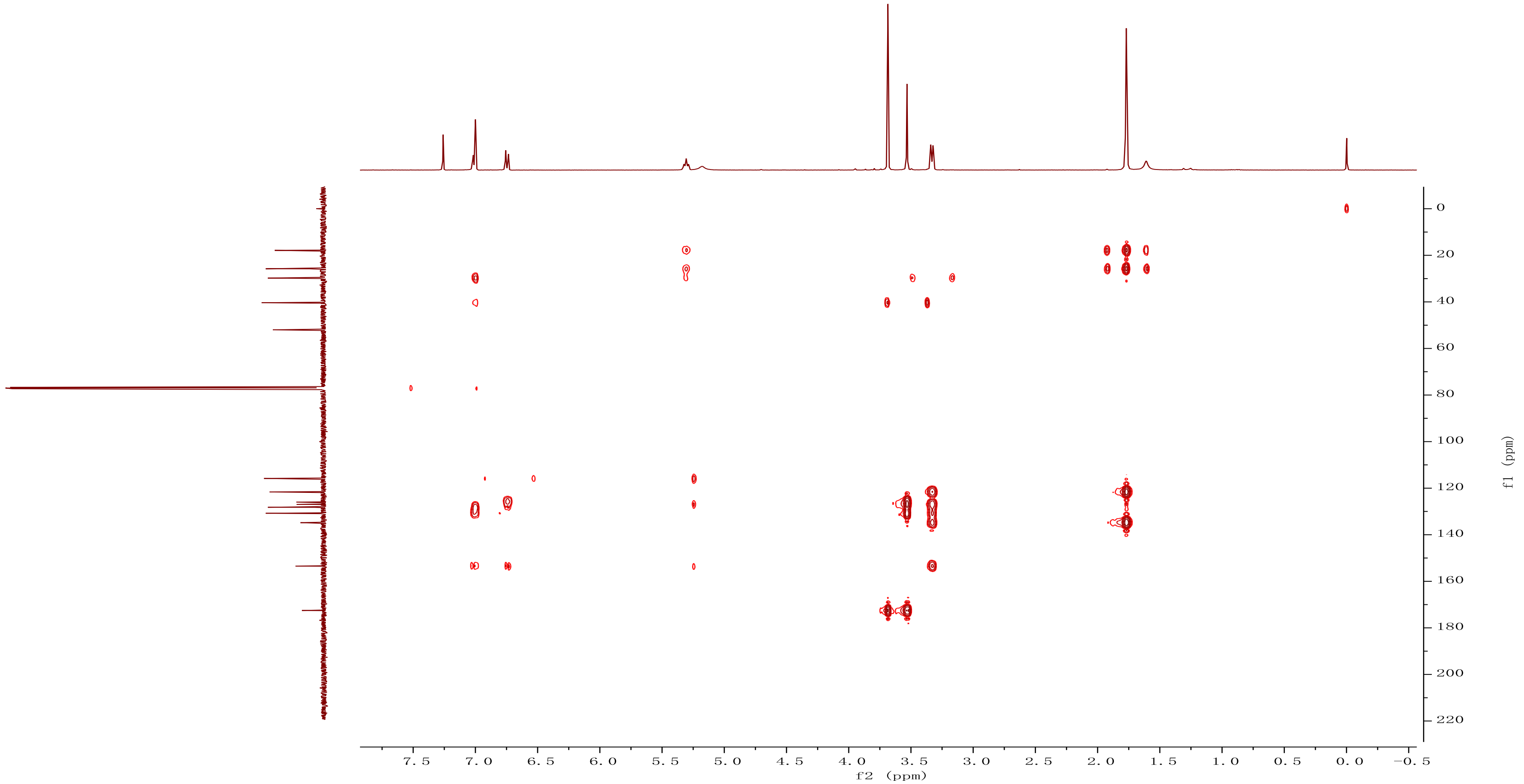

Figure S20. ^{1}H–^{1}H COSY spectrum of asperulosin C (**3**) in $CDCl_3$.

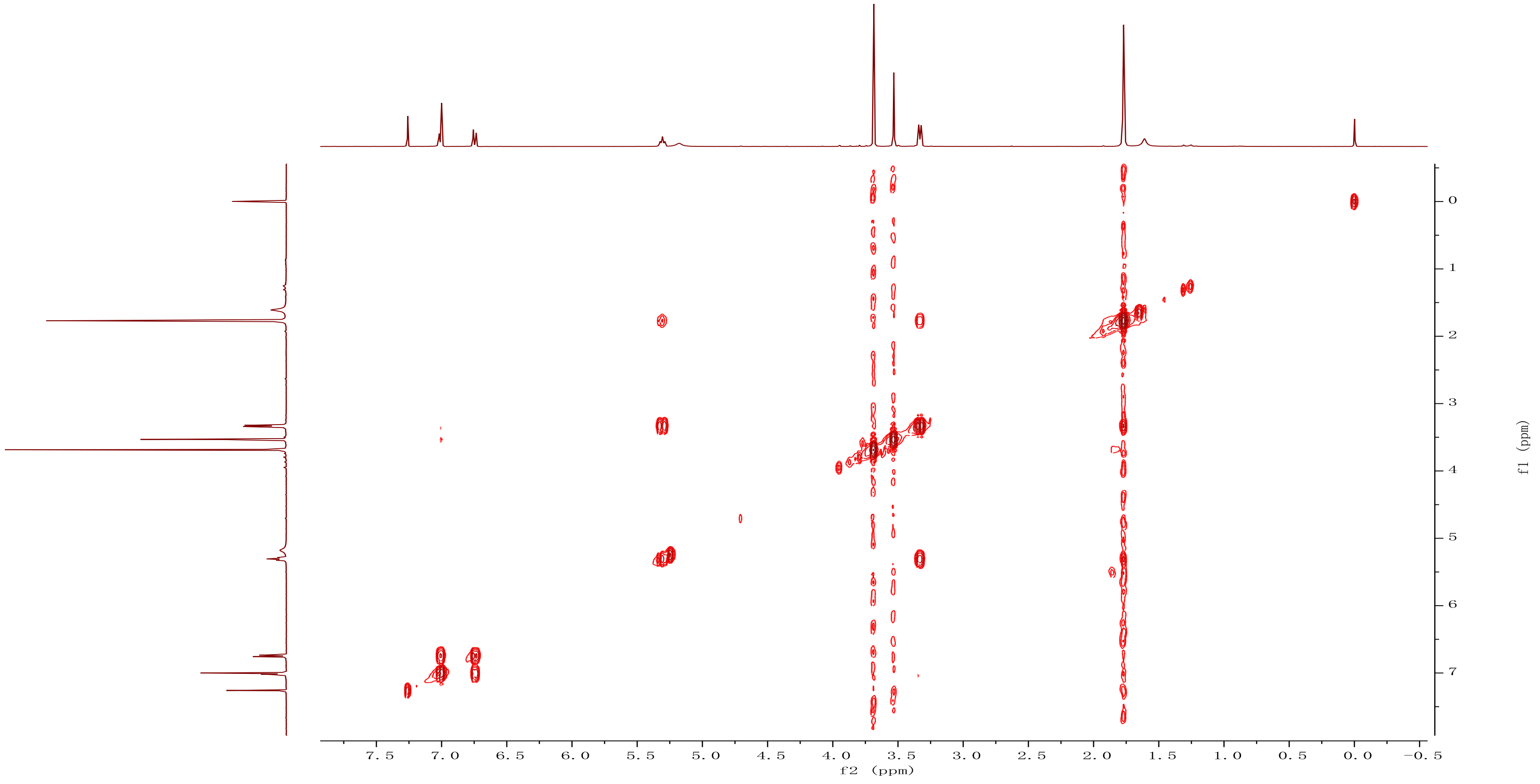

Figure S21. HRESIMS spectrum of asperulosin C (3).

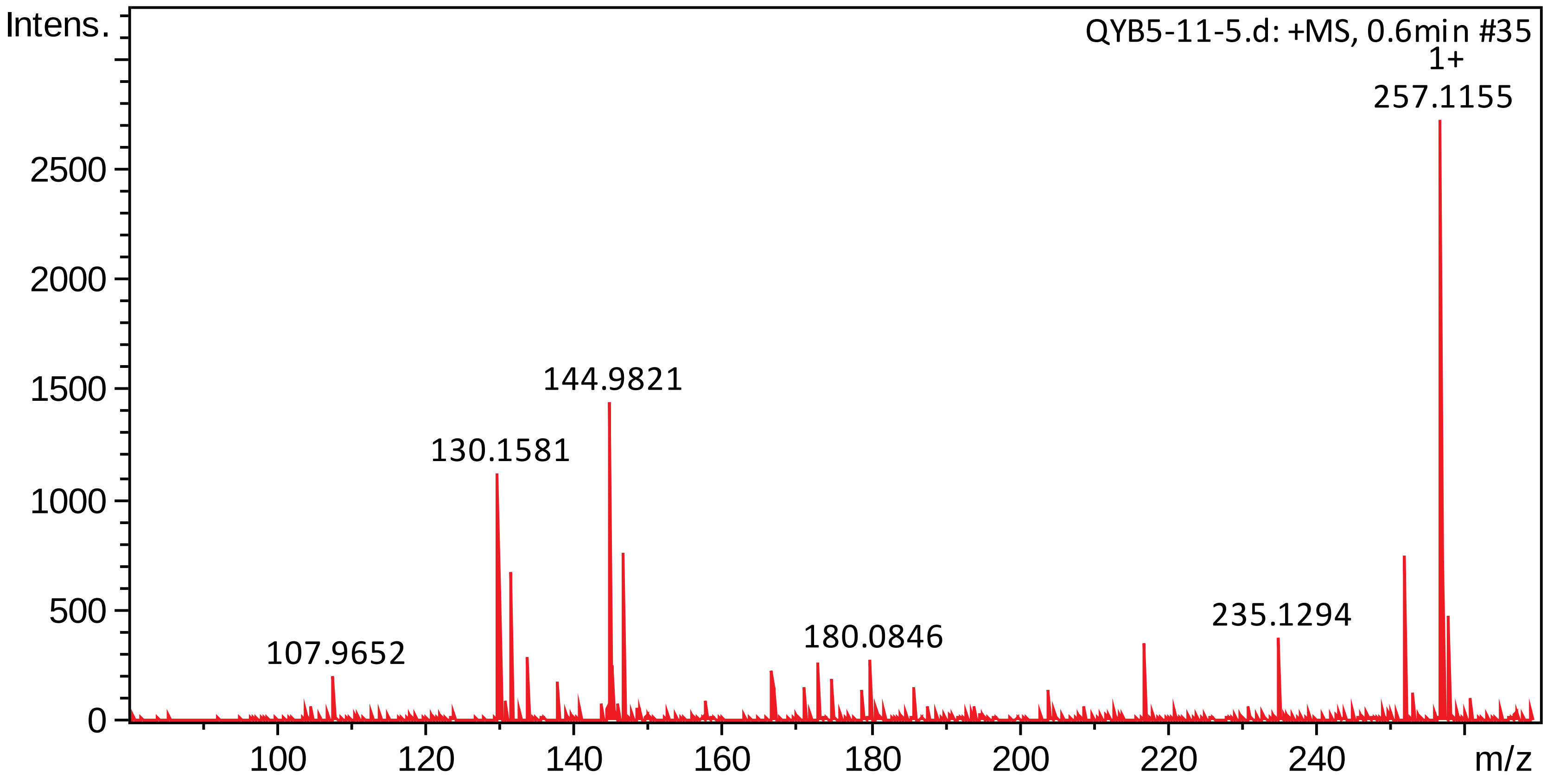

Figure S22. IR spectrum of asperulosin C (**3**).

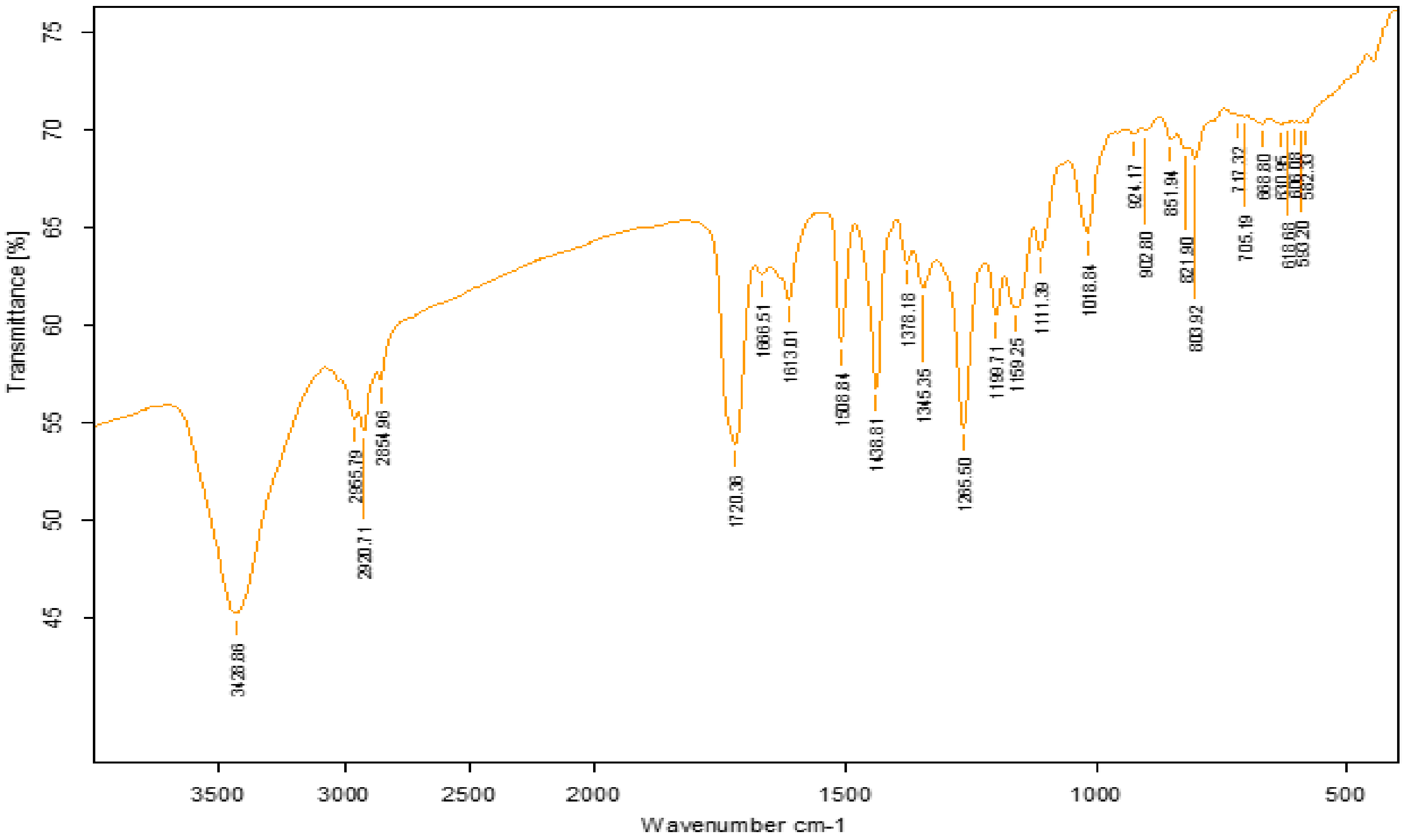

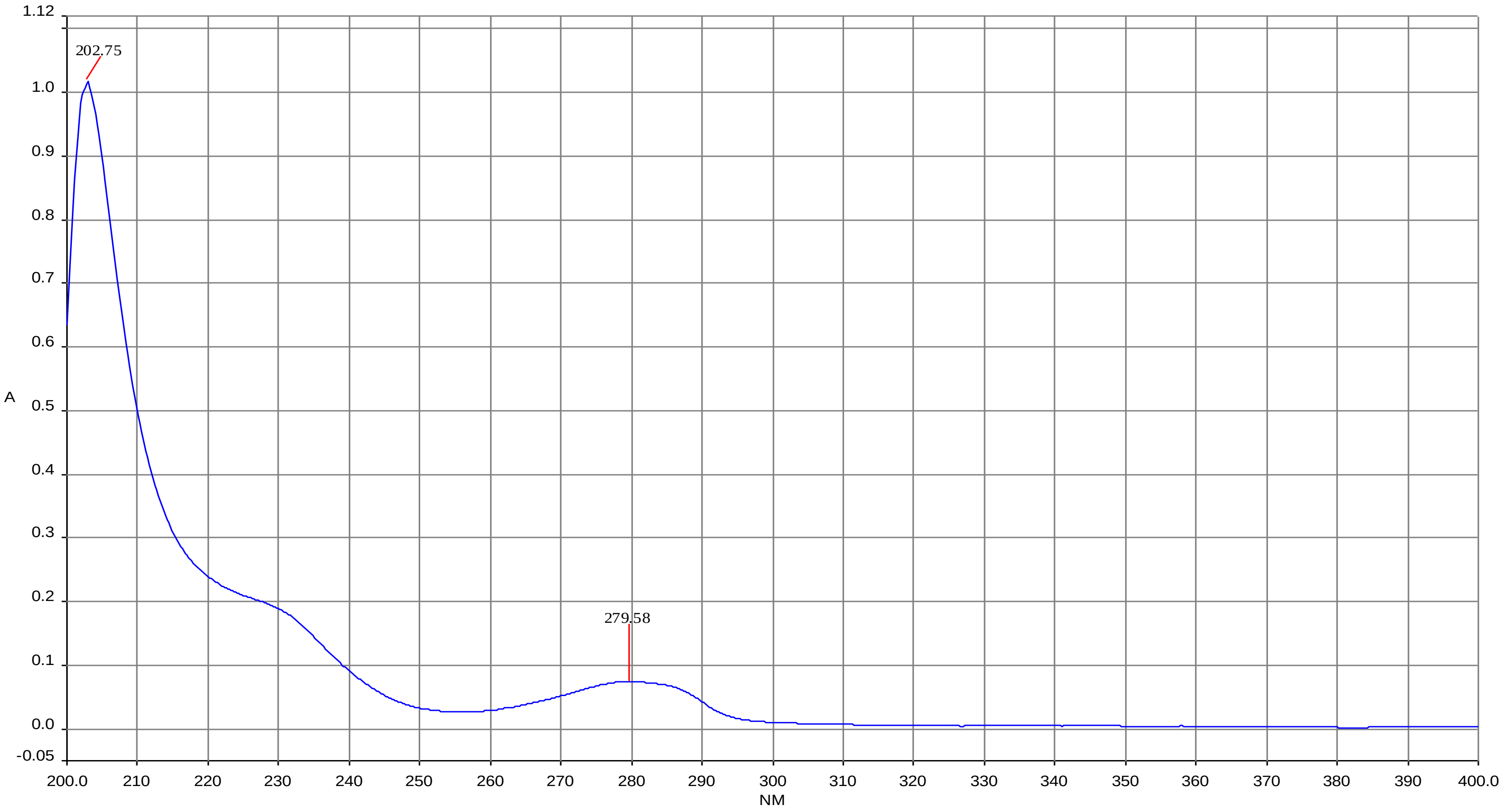

Figure S23. UV spectrum of asperulosin C (**3**).

Table S1. The corresponding primer sequences used in the experiment.

Mouse gene	Sense	Antisense
IL1β	CTACCTGTGTCTTTCCCGTG	TTTGTTGTTCATCTCGGAGC
IL6	TAAAATAGTCCTTCCTACCCC	TTGCCGAGTAGATCTCAAA
IL10	CAACATACTGCTAACCGACT	CTGGATCATTTCCGATAAGGC
TNF-α	CGTCGTAGCAAACCACCAAGT	CCATCGGCTGGCACCACTA
ARG1	CTGGCCTTTGTTGATGTCCCT	CCAGCACCACACTGACTCTTC
CD206	ATCCACGAGCAAATGTACCTCA	TAGCCAGTTCAGATACCGGAA
iNOS	TTGGCTCCAGCATGTACCCTC	TGCTTCGGACATCAAAGGTCT
COX-2	AGAAGGAAATGGCTGCAGAA	GCTCGGCTTCCAGTATTGAG
β-actin	CGTGCGTGACATCAAAGAGAA	TGGATGCCACAGGATTCCAT
